# Supplementary material for: Hydroxymethanesulfonate formation accelerated at the air-water interface by synergistic enthalpy-entropy effects
Source: Nat Commun. 2025 Jun 4;16:5187. doi: 10.1038/s41467-025-59712-3 (PMC12137664; doi:10.1038/s41467-025-59712-3)
Supplement: Supplementary file 1 — Supplementary Information [file 41467_2025_59712_MOESM1_ESM.pdf]

Supplementary Information

Hydroxymethanesulfonate Formation Accelerated at the Air-water Interface by Synergistic Enthalpy-Entropy Effects

Jifan Li[1,#], Weiqiang Tang[2,#], Jiabao Zhu[1], Jinrong Yang[1,*], and Xiao He[1,3,4,*]

[1]Shanghai Engineering Research Center of Molecular Therapeutics and New Drug Development, Shanghai Frontiers Science Center of Molecule Intelligent Syntheses, School of Chemistry and Molecular Engineering, East China Normal University, Shanghai, 200062, China;

[2]School of Chemical Engineering, East China University of Science and Technology, Shanghai, 200237, China

[3]Chongqing Key Laboratory of Precision Optics, Chongqing Institute of East China Normal University, Chongqing 401120, China

[4]New York University–East China Normal University Center for Computational Chemistry, New York University Shanghai, Shanghai, 200062, China

[#]These authors contribute equally to this work

Email: jryang@chem.ecnu.edu.cn; xiaohe@phy.ecnu.edu.cn

Contents

Supplementary Note 1. General workflow of this study. 1

Supplementary Figure 1 General workflow of this study. The General workflow contains the reaction simulation (left) and mechanism studies (right) of the nucleophilic addition between HCHO and $HOSO_2^-/SO_3^{2-}$. Metadynamics-biased AIMD simulation is conducted as a powerful tool to generate the free-energy profiles of the reaction. High-level quantum chemical calculation, Rx-DFT calculation and wavefunction analysis are carried out to analyse the influence of the external electric field and water molecules. In addition, the classical MD simulations are carried out as a supplementary investigation to analyse the interfacial stabilization. 2

Supplementary Note 2. Details of metadynamics-biased AIMD simulation. 3

2.1 Preparation of the simulation system 3

2.2 Energy minimization and pre-equilibrium 3

2.3 Metadynamics-biased AIMD simulation 3

Supplementary Figure 2. Workflow of the metadynamics-biased AIMD simulation. 5

Supplementary Figure 3. Three reaction pathways of metadynamics-biased AIMD simulations. Pathway 1 and 4 describe the nucleophilic addition of HCHO + $HOSO_2^-/HSO_3^-$ reaction without constraint the proton transfer process. Pathway 2 describe the nucleophilic addition of HCHO + $HOSO_2^-$ reaction by constraining a water molecule participating the proton transfer. Pathway 3 describe the nucleophilic addition of HCHO + SO_3^{2-} reaction. 6

Supplementary Table 1. Schematic representation and the definition of CV(s) in metadynamics-biased AIMD simulation of the following reaction pathways. 7

Supplementary Table 2. Simulation results of the metadynamics-biased AIMD simulation, the simulation time of each independent simulation, the CV values of the stationary points on the free-energy profiles and their corresponding Gibbs free-energies. 8

Supplementary Table 3. Details of the parameters of the metadynamics-biased AIMD simulation. 9

Supplementary Figure 4. Metadynamics-biased results for pathway 1 in bulk solution. (a) Gibbs free-energy profiles as a function of CV changes. **(b)** CV changes as a function of simulation time for three independent simulations. 10

Supplementary Figure 5. Metadynamics-biased results for pathway 1 at the air-water interface. (a) Gibbs free-energy profiles as a function of CV changes. **(b)** CV changes as a function of simulation time for three independent simulations..11

Supplementary Figure 6. (a) Definition of the air-water interface for the HCHO + $HOSO_2^-$ reaction (pathway 1) with 192 water molecules. **(b)** Gibbs free-energy profiles as a function of CV changes. **(c)** CV evolution as a function of simulation time. **(d)** Snapshot structures (**R_{inter}**, **TS_{inter}** and **P_{inter}**) obtained from the AIMD trajectories. 12

Supplementary Figure 7. (a) Gibbs free-energy profiles as a function of CV_1 **(b)** Evolution of CV_1 as a function of simulation time. **(c)** Evolution of CV_2 as a function of simulation time. **(d)** Snapshot structures (**R**, **TS** and **P**) obtained from the AIMD trajectories. 13

Supplementary Figure 8. Metadynamics-biased results for pathway 3 in bulk solution. (a) Gibbs free-energy profiles as a function of CV changes. **(b)** CV changes as a function of simulation time for three independent simulations. 14

Supplementary Figure 9. Metadynamics-biased results for pathway 3 at the air-water interface. (a) Gibbs free-energy profiles as a function of CV changes. **(b)** CV changes as a function of simulation time for three independent simulations..15

Supplementary Figure 10. (a) Variation of the CV (Top) and the angle between the S−C direction vector and the *z*-axis (Bottom) as a function of the simulation time. The purple shaded area represents the transition state, the light blue area represents the angle for every frame, the dark blue line in the bottom figure is the smoothed value (2500 points) to clarify the trend. **(b)** Snapshot of the $\theta = 37.09°$ at the air-water interface in 77.17 ps. 16

Supplementary Figure 11. (a) Variation of C···O (red line) and S···O (blue line) distances as a function of the simulation time. Shaded area is the distance of every frame, bold lines is the smoothed value (500 points) to clarify the trend. **(b)** Variation of the angle between the C=O direction vector and the *z*-axis (θ_1, 0–180°, red line) and the dihedral angle between C=O direction vector and the S−OH direction vector (θ_2, 0–180°, blue line) as a function of the simulation time. Shaded area is the angle of every frame, bold lines is the smoothed value (2000 points) to clarify the trend. **(d)** (Left) Relevant O···H distance differences (D_1–D_4) variation as a function of the simulation time. (Right) Schematic representation of the water-mediated proton transfer in bulk solution, the proton-roaming pathway is represented as red dash lines. **(d)** Snapshot of the water-mediated proton transfer extracted from the AIMD trajectories of the of HCHO + $HOSO_2^-$ aqueous reaction...17

Supplementary Note 3. Electron density difference analysis of the transition state of reaction 1 at the air water interface. 18

Supplementary Figure 12. Isodensity surface (isodensity = 0.02) of the distribution of the density difference of heterogeneous HCHO + $HOSO_2^-$ reaction. Green denotes regions of positive values and blue regions of negative values. 19

Supplementary Note 4. Details of quantum chemical calculations for the HCHO + $HOSO_2^-$ + $(H_2O)_n$ (n = 0, 1, 2, 3, 4) reactions, the HCHO + SO_3^{2-} + $(H_2O)_n$ (n = 0, 1, 2, 3, 4) reactions and corresponding wave function analysis...20

4.1 Geometrical optimization and the conformational search of global minimum.................20

4.2 High-level quantum chemical calculations 20

4.3 Charge composition analysis (CDA)..20

Supplementary Note 5. Details of quantum chemical calculations for the HCHO + $HOSO_2^-$ and HCHO + SO_3^{2-} reactions with and without an external electric field of 0.1 V/Å...............21

5.1 The calculation of the direction of the external electric field..21

5.2 Calculation the energy barrier by adding an electric field 21

Supplementary Figure 13. Workflow of the high-level quantum chemical calculation of the reaction complexes of the HCHO + $HOSO_2^-$ + $(H_2O)_n$ (n = 0, 1, 2, 3, 4) reaction and the HCHO + SO_3^{2-} + $(H_2O)_n$ (n = 0, 1, 2, 3, 4) reaction. 22

Supplementary Figure 14. Workflow of the high-level quantum chemical calculation of the transition states of the HCHO + $HOSO_2^-$ + $(H_2O)_n$ (n = 0, 1, 2, 3, 4) reaction and the HCHO + SO_3^{2-} + $(H_2O)_n$ (n = 0, 1, 2, 3, 4) reaction...23

Supplementary Figure 15. Workflow of the high-level quantum chemical calculation of the products of the HCHO + $HOSO_2^-$ + $(H_2O)_n$ (n = 0, 1, 2, 3, 4) reaction and the HCHO + SO_3^{2-} + $(H_2O)_n$ (n = 0, 1, 2, 3, 4) reaction...24

Supplementary Figure 16. Workflow of the High-level quantum chemical calculations for the HCHO + $HOSO_2^-$ and HCHO + SO_3^{2-} reactions with and an external electric field of 0.1 V/Å. 25

Supplementary Figure 17. The dipole moment vector (blue) and electric field strength vector (black) of transition states for the reactions: **(a)** HCHO + $HOSO_2^-$, **(b)** HCHO + SO_3^{2-}. 26

Supplementary Figure 18. (a) Gibbs free-energy profiles of the HCHO + $HOSO_2^-$ reaction with the addition of H_2O molecules (HCHO + $HOSO_2^-$ + $(H_2O)_n$, n = 0, 1, 2, 3, 4) and the addition of an external electric field of 0.1 V/Å (**R1**$_E$, **TS1**$_E$ and **P1**$_E$). **(b)** Corresponding structures of the stationary points optimized at the M06-2x/6-311++G(d, p) level of theory, relative single point

energies of reactant complexes, transition states, and products were obtained at the CCSD(T)/aug-cc-PVTZ level of theory. 27

Supplementary Figure 19. (a) Gibbs free-energy profiles of the HCHO + SO_3^{2-} reaction with the addition of H_2O molecules (HCHO + SO_3^{2-} + $(H_2O)_n$, n = 0, 1, 2, 3, 4) and the addition of an external electric field of 0.1 V/Å (**R2**$_E$, **TS2**$_E$ and **P2**$_E$). **(b)** Corresponding structures of the stationary points optimized at the M06-2x/6-311++G(d, p) level of theory, relative single point energies of reactant complexes, transition states, and products were obtained at the CCSD(T)/aug-cc-PVTZ level of theory. 28

Supplementary Figure 20. Calculated Laplacian bond order of **TS1 (a)** and **TS1**$_E$ **(b)**. 29

Supplementary Note 6. Interaction free-energy of HMS-H_2O, HMS-SO_2, and HMS-$HOSO_2^-$ complexes. 30

Supplementary Figure 21. Optimized structures of HMS. **(a)**, HMS−H_2O **(b)**, HMS−SO_2 **(c)** and HMS−$HOSO_2^-$ **(d)** with M06-2x/6-311++G(d,p) and PCM model with water solvent. 31

Supplementary Table 4. Calculated ΔG_{com} of different complexes. 32

Supplementary Note 7. Details of CMD simulations. 34

7.1 Preparation of the simulation system 34

7.2 Details of CMD parameters 34

7.3 Simulation of HCHO@$(H_2O)_{1000}$ system, pure water, and HCHO saturated solution. 34

7.4 Umbrella sampling techniques 35

Supplementary Table 5. The molecules, the parameters of the simulation box and the simulation time of CMD simulation. 36

Supplementary Note 8. Definition of the angle between the dipole vector and the *x*(a), *y*(b), *z*(c) axis. 37

Supplementary Note 9. Details of the calculation of the dipole autocorrelation function. 38

Supplementary Note 10. Details of the simulation of the HCHO saturated solution at the air-water interface. 39

Supplementary Figure 23. (a) The schematic plot of the system for simulating the HCHO saturated solution at the air-water interface. **(b)** The density of mass (black line) of the HCHO saturated solution and the molecular number density of HCHO (red line) as a function of *z*-position. 40

Supplementary Figure 24. The schematic plot of windows the US techniques. 41

Supplementary Figure 25. Gibbs free-energy profile and the schematic plot of the system for calculating a SO_3^{2-} molecule from the bulk water across the air-water interface into the gas phase. 42

Supplementary Note 11. Details of the calculation of the activation entropy of the proton transfer. 43

Supplementary Note 12. Investigation of the influence of inorganic ion. 44

Supplementary Figure 26. The reaction between $HOSO_2^-$ and HCHO in the bulk salt solution. (a) Snapshot structures (reactant, $\mathbf{R_{ion}}$, transition state, $\mathbf{TS_{ion}}$ and product, $\mathbf{P_{ion}}$) obtained from the metadynamics-biased AIMD simulations. The purple ball indicates the Na^+ ion and the green ball represents the Cl^- ion. The water molecules coordinated to the Na^+ ion are marked as the licorice style for clarity. **(b)** Gibbs free-energy profile as a function of collective variable (CV). **(c)** (Left) Temporal evolution of the distance between the Na^+ ion and the COM (star) of reactants (D_{ion}, purple), along with the variation of the CV (blue). (Right) Schematic definition of D_{ion} and the CV. 46

Supplementary Note 13. Investigation of the reactivity of sulfonate. 47

Supplementary Figure 27. Accelerated reactivity of the sulfonate (HSO_3^-) to form the HMSi formation at the air-water interface. (a) Electrostatic potential surface of HSO_3^-. Red regions denote positive electrostatic potential and blue regions of negative potential. **(b)** Isovalue surfaces of the highest occupied molecular orbital (HOMO) of the HSO_3^- (isovalue = ±0.005). **(c)** Representative reaction mechanism for HCHO + HSO_3^- reaction, where the HSO_3^- functions as the nucleophile to attack the carbonyl group. **(d)** Gibbs free-energy profiles for HCHO + HSO_3^- reaction in gas phase with their corresponding optimized structures of the stationary points (**R**, **INT**, **TS** and **P**) at the M06-2x/6-311++G(d,p) level of theory. **(e)** Gibbs free-energy profile of the HCHO + HSO_3^- reaction at the air-water interface obtained from TI-AIMD simulation with the average values of each window (blue dots) for three independent simulations, relevant error bars (colored orange) of each window are calculated using the standard deviation of the corresponding free-energy values of three production runs. The free-energy profile (red line) is generated by Piecewise Cubic Hermite Interpolating Polynomial (PCHIP) interpolation of calculated average free-energy values of three production runs for each window. **(f)** (Top) Definition of the CV for the TI-AIMD simulation. (Bottom) Snapshot structures (reactant, $\mathbf{R_{sul}}$, transition state, $\mathbf{TS_{sul}}$ and product, $\mathbf{P_{sul}}$) obtained from the TI-AIMD simulations. 48

Supplementary Note 14. Influence of Aerosol Acidity or pH. 49

Supplementary Figure 28. Reaction pathways concerning S(IV) species under extreme acidic conditions (pH = 0.8–1.8), the abundant presence of sulfonate (HSO_3^-) enables its reaction with surface-accumulated HCHO to form HMSi. 50

Supplementary Figure 29. Reaction pathways concerning S(IV) species from weak acidic to neutral organic aerosols (pH > 4). The concentration sulfite (SO_3^{2-}) augments due to the deprotonation of the sulfonate (HSO_3^-) and the bisulfite ($HOSO_2^-$). The SO_3^{2-} reacts with HCHO at the air-water interface or in the bulk solution to form the HMS. 51

Supplementary Note 15. Details of the thermodynamic integration (TI)-AIMD simulation. . 52

Supplementary Figure 30. TI-AIMD simulation of the reaction between SO_3^{2-} and HCHO in the bulk solution. (a) Gibbs free-energy profile obtained from TI-AIMD simulation with the average values of each window (blue dots) of three independent simulations, relevant error bars (colored orange) of each window are calculated using the standard deviation of the corresponding free-energy values of three production runs. The free-energy profile (purple line) is generated by Piecewise Cubic Hermite Interpolating Polynomial (PCHIP) interpolation of calculated average free-energy values of three production runs. **(b)** (Top) Definition of the CV for the TI-AIMD simulation. (Bottom) Snapshot structures (reactants: $\mathbf{R_{TI}}$, transition state: $\mathbf{TS_{TI}}$ and product: $\mathbf{P_{TI}}$) obtained from the trajectories of TI-AIMD simulation. 53

Supplementary References 54

Supplementary Note 1. General workflow of this study.

The general workflow consists of two major parts. The representative workflow is shown in the **Supplementary Figure 1**. On the one hand, the aqueous and heterogeneous reaction are simulated by using metadynamics-biased[1] ab initio molecular dynamics (AIMD) simulation to calculate Gibbs free-energy profile for HCHO + $HOSO_2^-$ and HCHO + SO_3^{2-} reactions in bulk water and at the air-water interface, respectively and the HCHO + $HOSO_2^-$ reaction in the bulk salt solution. On the other hand, high-level quantum chemical calculations, classic molecular dynamics (CMD) simulations and Reaction Density Functional Theory (Rx-DFT) calculations combined with wave function analysis are employed to reveal the quantitative mechanism of HMS formation. In addition, thermodynamic integration (TI)-AIMD simulation were carried out to investigate the reaction of HCHO + HSO_3^- at the air-water interface and validate the metadynamics results for the SO_3^{2-} + HCHO reaction in the bulk solution.

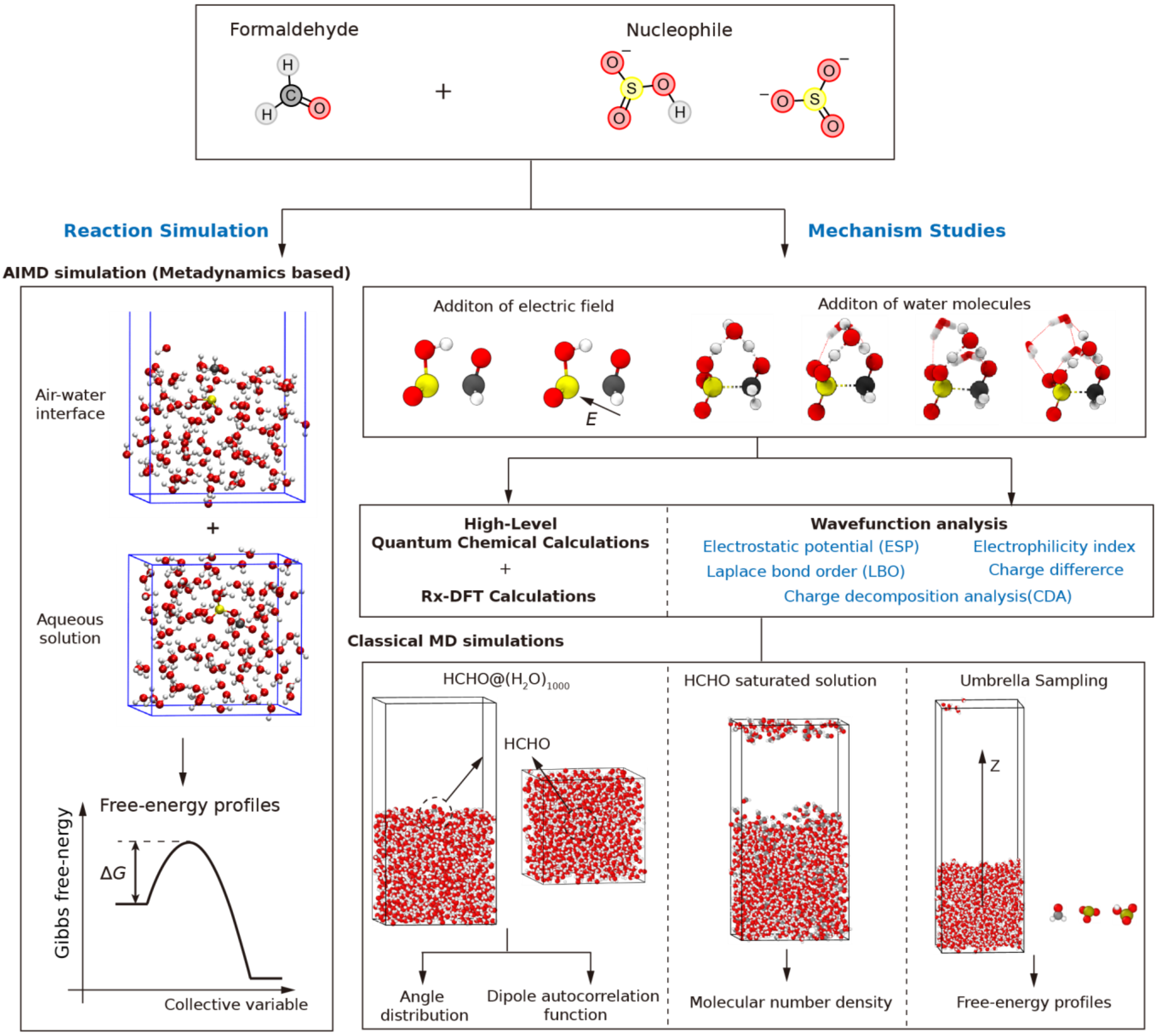

Supplementary Figure 1 General workflow of this study. The General workflow contains the reaction simulation (left) and mechanism studies (right) of the nucleophilic addition between HCHO and $HOSO_2^-/SO_3^{2-}$. Metadynamics-biased AIMD simulation is conducted as a powerful tool to generate the free-energy profiles of the reaction. High-level quantum chemical calculation, Rx-DFT calculation and wavefunction analysis are carried out to analyse the influence of the external electric field and water molecules. In addition, the classical MD simulations are carried out as a supplementary investigation to analyse the interfacial stabilization.

Supplementary Note 2. Details of metadynamics-biased AIMD simulation.

2.1 Preparation of the simulation system

The cartesian coordinates of the stationary structure of HCHO, H_2O, and $HOSO_2^-$, SO_3^{2-} molecules were firstly optimized by using the Gaussian 16 program at the M06-2x/6-311G(d,p) and M06-2x/6-311G++(d,p) level respectively[2-7]. All simulation systems are carried out for a serious of bulk solution and air-water interface systems, constructed of the reactants (HCHO + $HOSO_2^-$/HCHO + SO_3^{2-}) with 96 water molecules and HCHO + $HOSO_2^-$ with a Na^+, a Cl^-, and 93 water molecules.

For pure bulk water system, the box size was converted from the density of ρ = 997 kg/m^3 of pure water at 300 K. For the NaCl solution, the concentrations of Na^+ and Cl^- equal to 0.577 mol/L. For the air-water interface, we used a water slab where the major reactants are placed on the surface firstly. To ensure the calculation accuracy, we simulated the heterogeneous reaction of HCHO + $HOSO_2^-$ with 192 H_2O molecules. All initial structures were firstly generated with the Packmol[8,9] program and all of the simulation box sizes settings are shown in **Supplementary Table 2**.

2.2 Energy minimization and pre-equilibrium

Both energy minimization and pre-equilibrium were performed using the cp2k software package[10]. We used the PM6[11] level to minimize the initial structure.

For pre-equilibrium, the Becke–Lee–Yang–Parr (BLYP) functional[12,13] with Grimme's[14] dispersion corrections and Becke-Johnson[15] damping term, and the double-ζ plus polarization (DZVP) basis set were used. An energy cutoff of 300 Ry was applied for the plane-wave basis set and 40 Ry cutoff was used for the Gaussian basis set. The core electrons were modeled using Goedecker–Teter–Hutter[16] (GTH) norm-conserving pseudopotentials. The AIMD simulations were performed in the canonical (NVT) ensemble, with a temperature of 298.15 K con-trolled using canonical sampling through velocity rescaling thermostat[17] (CSVR). The time step for was set for 1.0 fs. The pre-equilibrium process was terminated as the simulation temperature and the potential energy were well equilibrated.

2.3 Metadynamics-biased AIMD simulation

The AIMD parameters in the metadynamics-biased AIMD simulation are same as the previous part. For the metadynamics-biased AIMD simulation, we have calculated three different reaction pathways (see **Supplementary Figure 3**), the definition of each collective variables (CV) for each pathway are summarized in **Supplementary Table 1**. The details of associated parameters are summarized in **Supplementary Table 3**. Quadratic walls were applied to narrow the sampling space. The initial structure for the metadynamics-biased AIMD simulation were captured from unbiased AIMD

simulation trajectories. (Note that the initial value of the CV needs to be located in the sampling space). For the reaction pathway 1 and 3, three independent simulations with three Gibbs-energy profiles were performed in aqueous system and at the air water interface, respectively. The simulation results and the simulation time of each calculation, the CV values of the stationary points on the free-energy profiles and their corresponding Gibbs free-energies are summarized in **Supplementary Table 2**. Relevant error band of the free-energy profile are calculated on the standard deviation by employing cubic interpolation on three independently obtained free-energy profiles. The error of the free-energy barrier is the standard deviation of the free-energy barriers of three metadynamics simulations. All cp2k input files are firstly generated by using Multiwfn package[18]. The general work flow of the metadynamics-biased AIMD simulation is summarized in **Supplementary Figure 2**.

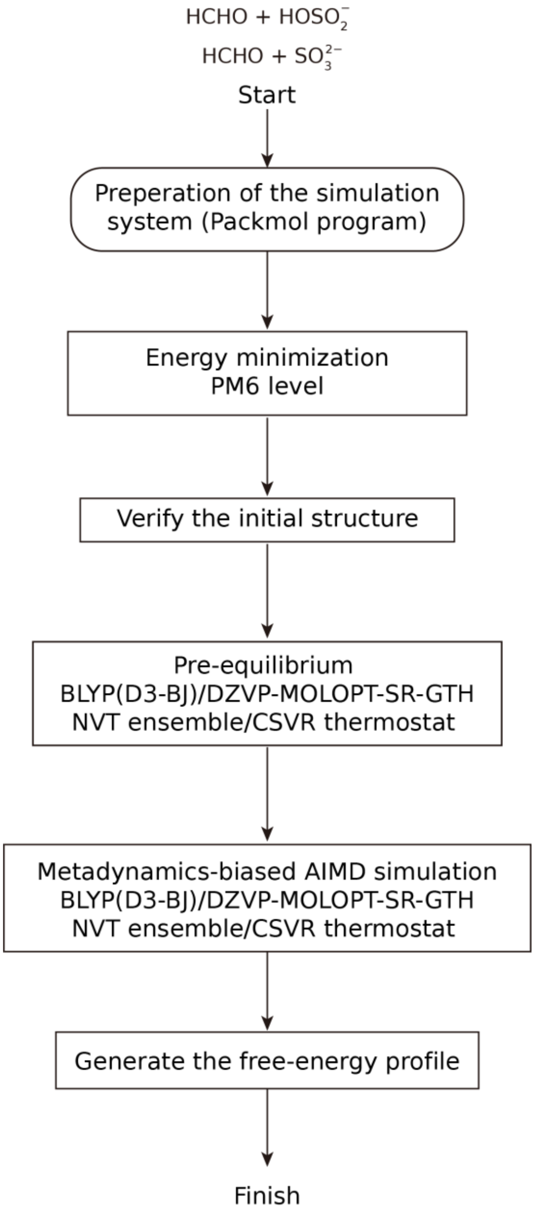

Supplementary Figure 2. Workflow of the metadynamics-biased AIMD simulation.

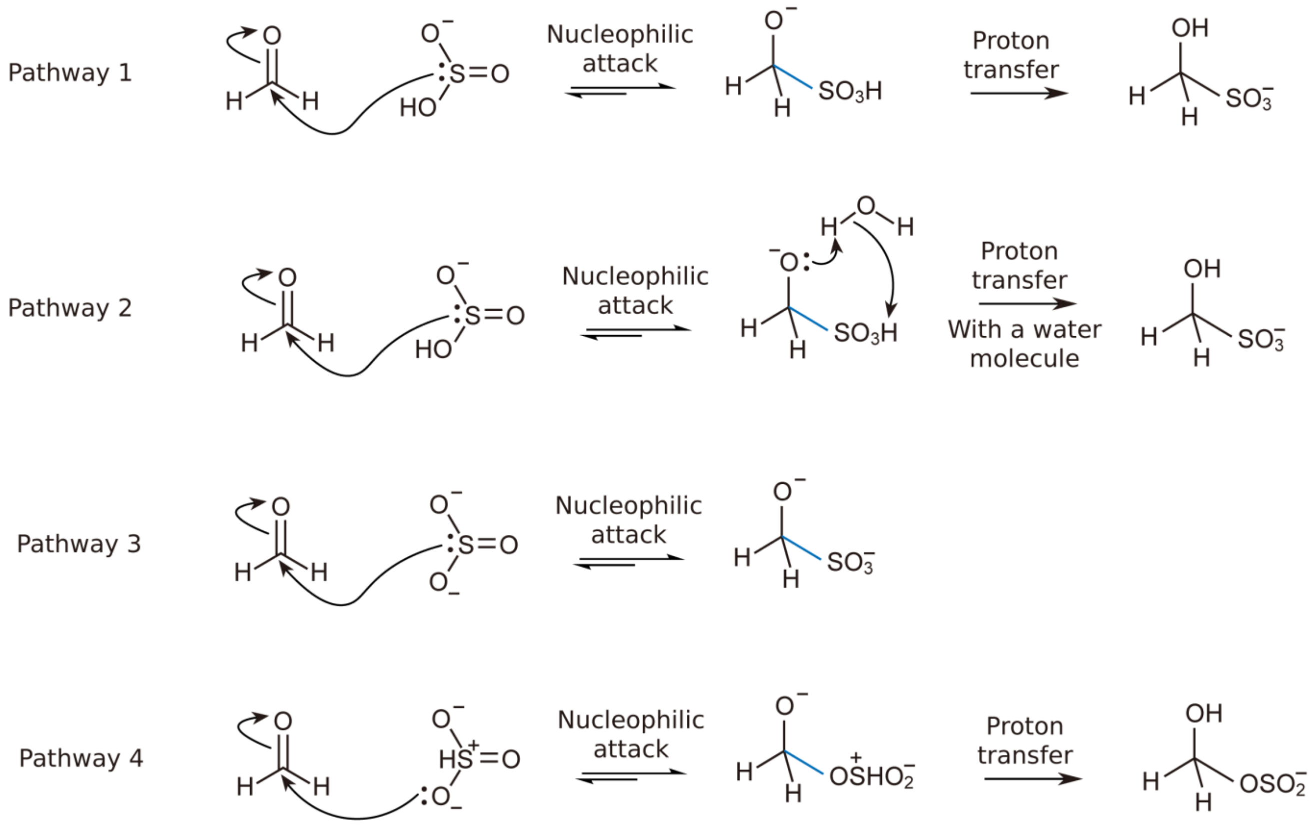

Supplementary Figure 3. Three reaction pathways of metadynamics-biased AIMD simulations. Pathway 1 and 4 describe the nucleophilic addition of HCHO + $HOSO_2^-$/HSO_3^- reaction without constraint the proton transfer process. Pathway 2 describe the nucleophilic addition of HCHO + $HOSO_2^-$ reaction by constraining a water molecule participating the proton transfer. Pathway 3 describe the nucleophilic addition of HCHO + SO_3^{2-} reaction.

Supplementary Table 1. Schematic representation and the definition of CV(s) in metadynamics-biased AIMD simulation of the following reaction pathways.

Reaction pathway	Schematic representation	Defined CV(s)
1	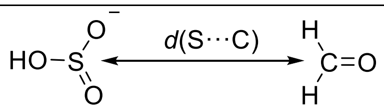	CV = d(S···C)
2	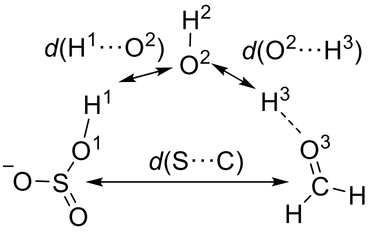	CV_1 = d(S···C) CV_2 = $d(H^1\cdots O^2)$ – $d(O^2\cdots H^3)$
3	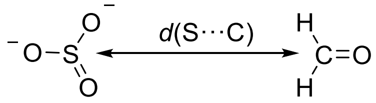	CV = d(S···C)

Supplementary Table 2. Simulation results of the metadynamics-biased AIMD simulation, the simulation time of each independent simulation, the CV values of the stationary points on the free-energy profiles and their corresponding Gibbs free-energies.

Pathway	Number	CV(R) (Å)	CV(TS) (Å)	CV(P) (Å)	*G*(R) (kcal/mol)	*G*(TS) (kcal/mol)	*G*(P) (kcal/mol)	Δ*G* (kcal/mol)[e]	ΔG_{avg} (kcal/mol)[f]	Simulation Time (ps)
1	1[a]	4.07	2.31	1.86	−24.4	−12.1	−25.5	12.3	11.4 ± 0.8	75
	2[a]	3.75	2.30	1.86	−19.7	−8.4	−19.0	11.3		55
	3[a]	3.33	2.24	1.83	−19.4	−8.7	−17.5	10.7		55
	1[b]	3.24	2.32	1.81	−23.2	−15.4	−25.2	7.7	7.6 ± 0.6	99
	2[b]	4.00	2.41	1.86	−16.4	−8.2	−19.2	8.2		65
	3[b]	4.00	2.24	1.86	−12.3	−5.4	−13.0	6.9		47.5
	[c]	4.02	2.29	1.88	−16.3	−9.8	−15.8	6.5		63
	[d]	3.71	2.20	1.94	−16.9	−6.7	−11.0	10.2		33
2	1[a]	3.95	2.46	1.87	−82.4	−3.6	−103.5	78.8		100
3	1[a]	3.49	2.33	1.90	−5.9	−0.2	−6.9	5.7	5.8 ± 0.1	21
	2[a]	3.25	2.34	1.86	−7.1	−1.4	−7.6	5.7		24
	3[a]	3.75	2.44	1.90	−7.8	−1.8	−8.5	5.9		28
	1[b]	3.92	2.28	1.89	−5.2	−1.7	−6.0	3.4	3.1 ± 0.5	38
	2[b]	3.22	2.32	1.93	−6.1	−3.5	−7.1	2.6		49
	3[b]	3.12	2.35	1.87	−9.0	−5.7	−10.9	3.3		75.5

[a]AIMD simulation in bulk solution, simulation box size: 1.42296 × 1.42296 × 1.42296 ($x \times y \times z$) nm^3.

[b]AIMD simulation at the air water interface, simulation box size: 1.5 × 1.5 × 3.0 ($x \times y \times z$) nm^3.

[c]AIMD simulation with $HOSO_2^-$ + HCHO @ $(H_2O)_{192}$ at the air the water interface, simulation box size: 1.5 × 1.5 × 6.0 ($x \times y \times z$) nm^3.

[d]AIMD simulation with $HOSO_2^-$ + HCHO + Na^+ + Cl^- @ $(H_2O)_{93}$ in bulk solution, simulation box size 1.42296 × 1.42296 × 1.42296 ($x \times y \times z$) nm^3.

[e] Energy barrier for each simulation is calculated as $G(\mathbf{TS}) - G(\mathbf{R})$.

[f] Average value and standard division of the reaction barrier from three independent metadynamics-biased AIMD simulations.

Supplementary Table 3. Details of the parameters of the metadynamics-biased AIMD simulation.

Pathway	Gaussian hill height (10^{-4} Hartree)	Gaussian hill width (Å)	Deposit time interval (fs)	CV_1 wall maximum[e]	CV_1 wall minimum	CV_2 wall maximum	CV_2 wall minimum
1[a]	1.8	0.08	30	7.5 Bohr	-	-	-
1[b]	1.114	0.1	30	7.5 Bohr	-	-	-
1[c]	1.114	0.1	30	7.5 Bohr	-	-	-
1[d]	2	0.1	30	4 Å	-	-	-
2[b]	1.114	0.1	30	7.5 Bohr	-	1.3 Bohr	−1.3 Bohr
3[a]	1	0.1	30	4 Å	-	-	-
3[a]	0.5	0.1	30	4 Å	-	-	-

[a]AIMD simulation in bulk solution.

[b]AIMD simulation at the air water interface.

[c]AIMD simulation with $HOSO_2^-$ + HCHO @ $(H_2O)_{192}$ at the air water interface.

[d]AIMD simulation with $HOSO_2^-$ + HCHO + Na^+ + Cl^- @ $(H_2O)_{93}$ in bulk solution.

[e] If the simulation has only one CV to define, the CV_1 represents to the individual CV.

[-] The value is not need to defined.

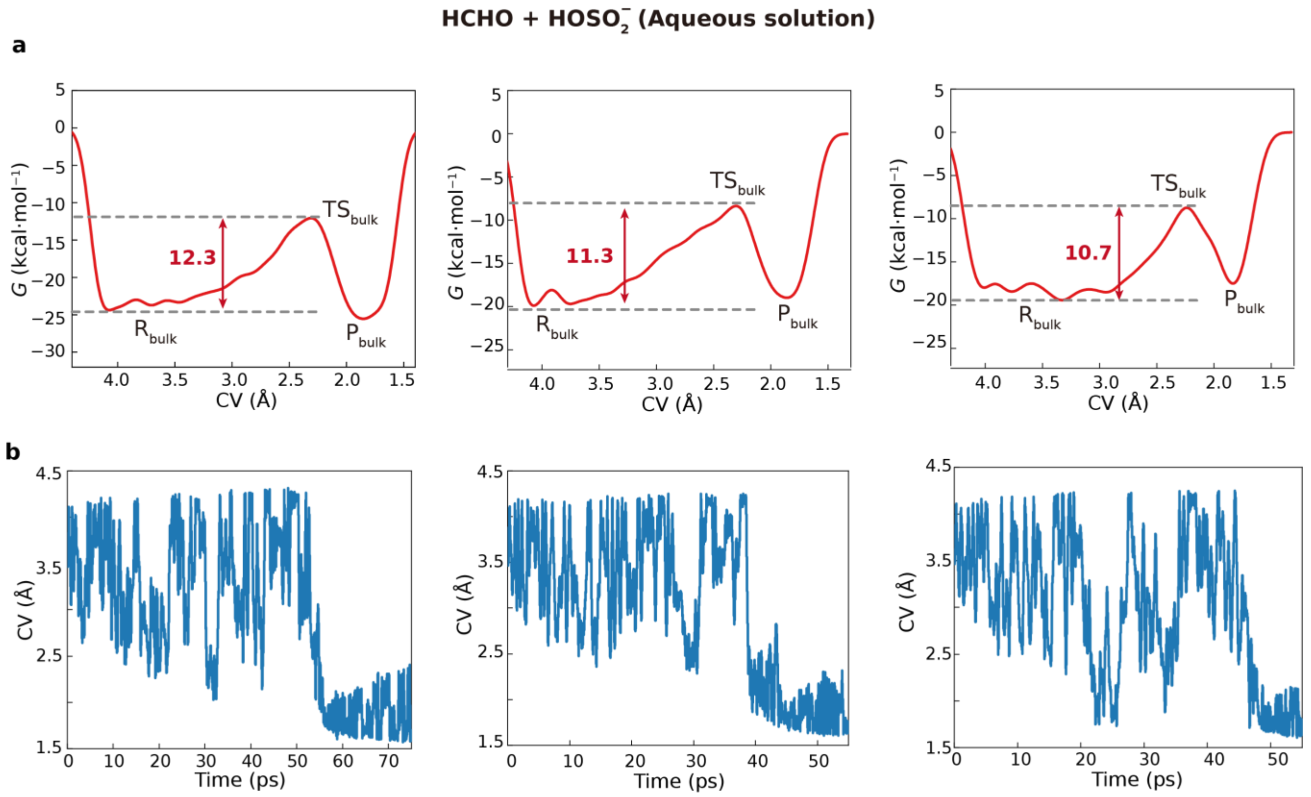

Supplementary Figure 4. Metadynamics-biased results for pathway 1 in bulk solution. (a) Gibbs free-energy profiles as a function of CV changes. **(b)** CV changes as a function of simulation time for three independent simulations.

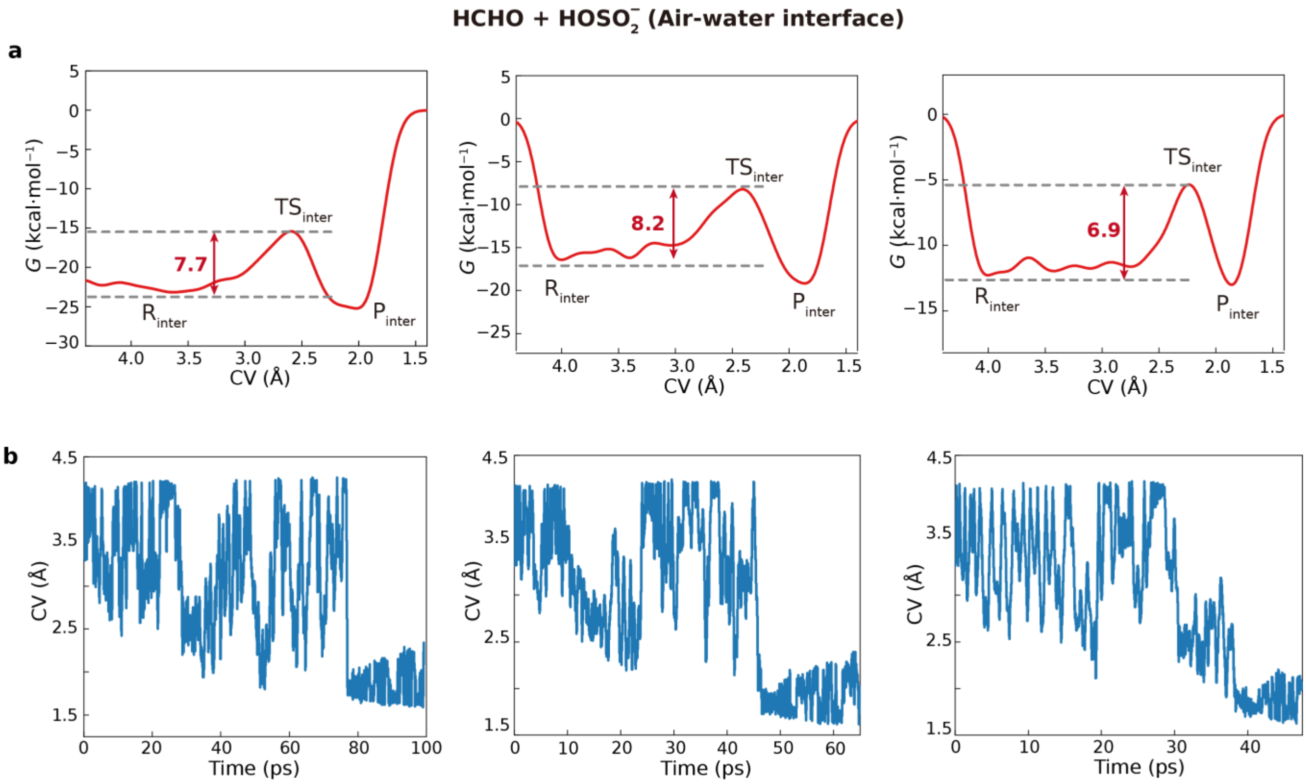

Supplementary Figure 5. Metadynamics-biased results for pathway 1 at the air-water interface. **(a)** Gibbs free-energy profiles as a function of CV changes. **(b)** CV changes as a function of simulation time for three independent simulations.

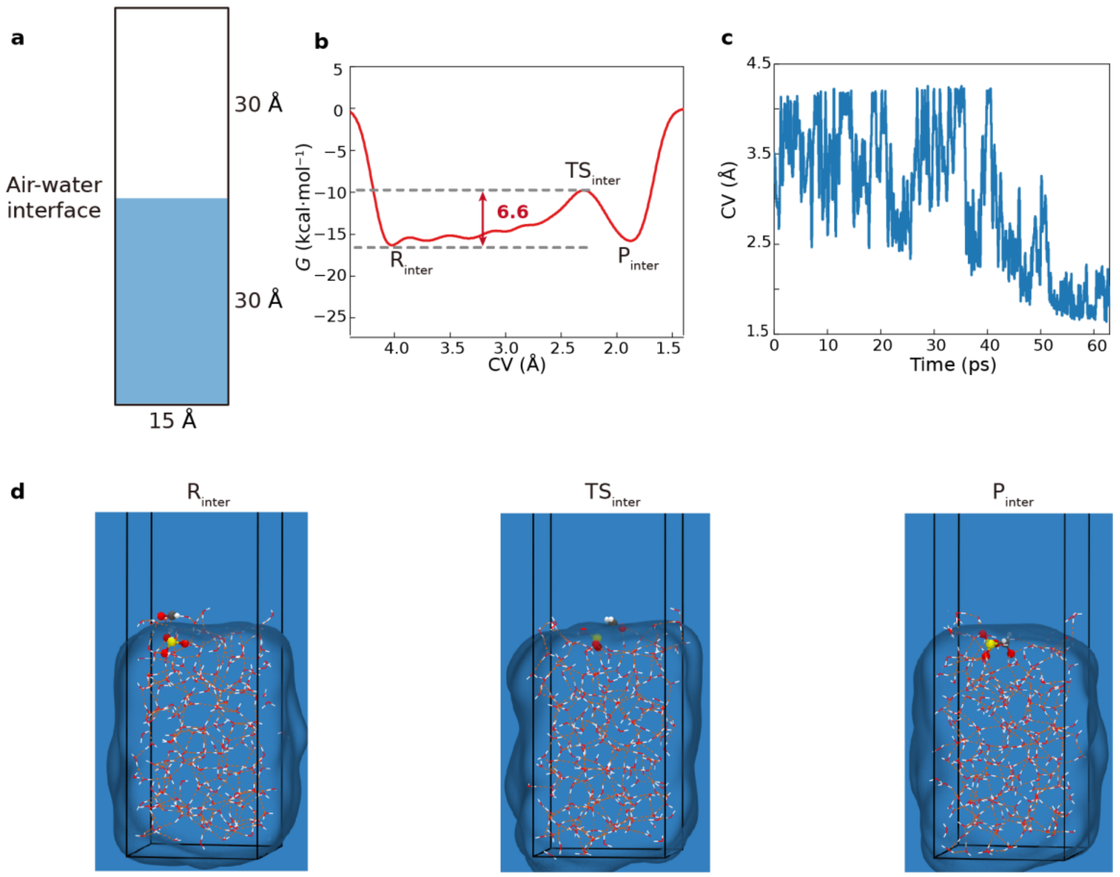

Supplementary Figure 6. (a) Definition of the air-water interface for the HCHO + $HOSO_2^-$ reaction (pathway 1) with 192 water molecules. **(b)** Gibbs free-energy profiles as a function of CV changes. **(c)** CV evolution as a function of simulation time. **(d)** Snapshot structures (**R$_{inter}$**, **TS$_{inter}$** and **P$_{inter}$**) obtained from the AIMD trajectories.

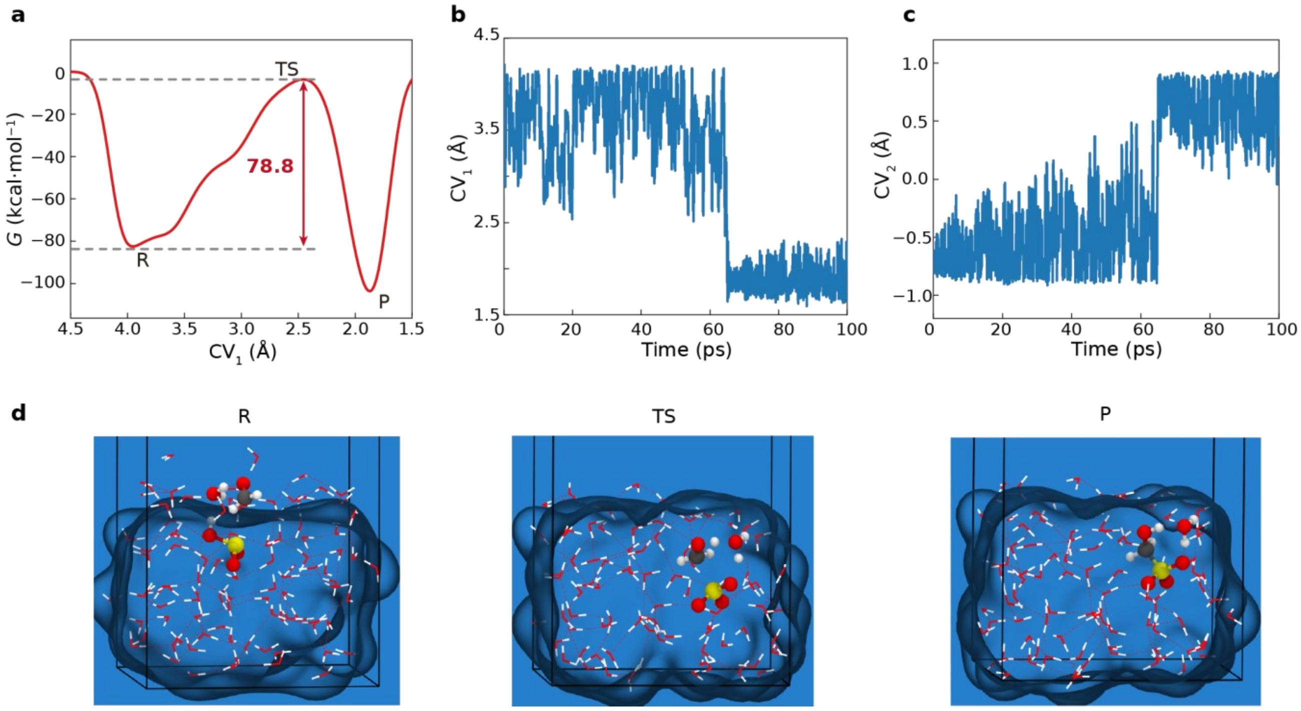

Supplementary Figure 7. (a) Gibbs free-energy profiles as a function of CV_1 **(b)** Evolution of CV_1 as a function of simulation time. **(c)** Evolution of CV_2 as a function of simulation time. **(d)** Snapshot structures (**R**, **TS** and **P**) obtained from the AIMD trajectories.

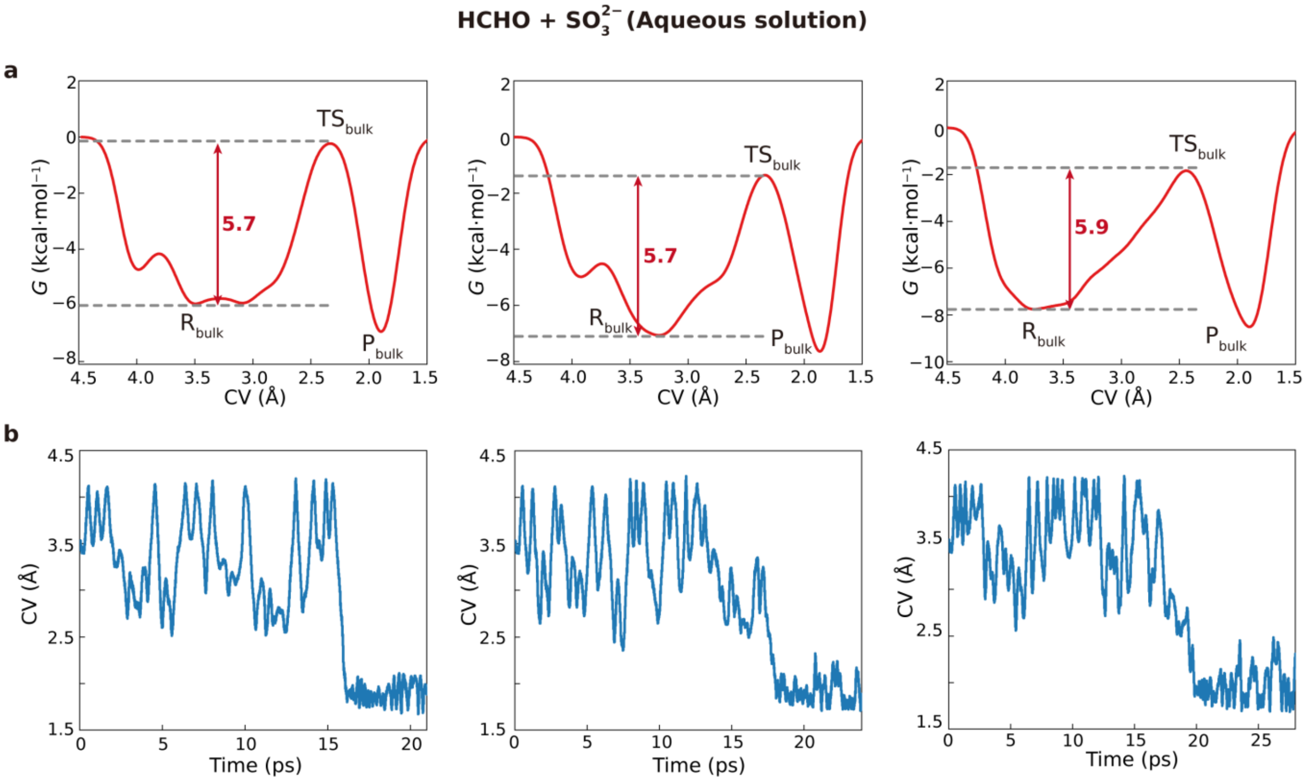

Supplementary Figure 8. Metadynamics-biased results for pathway 3 in bulk solution. (a) Gibbs free-energy profiles as a function of CV changes. **(b)** CV changes as a function of simulation time for three independent simulations.

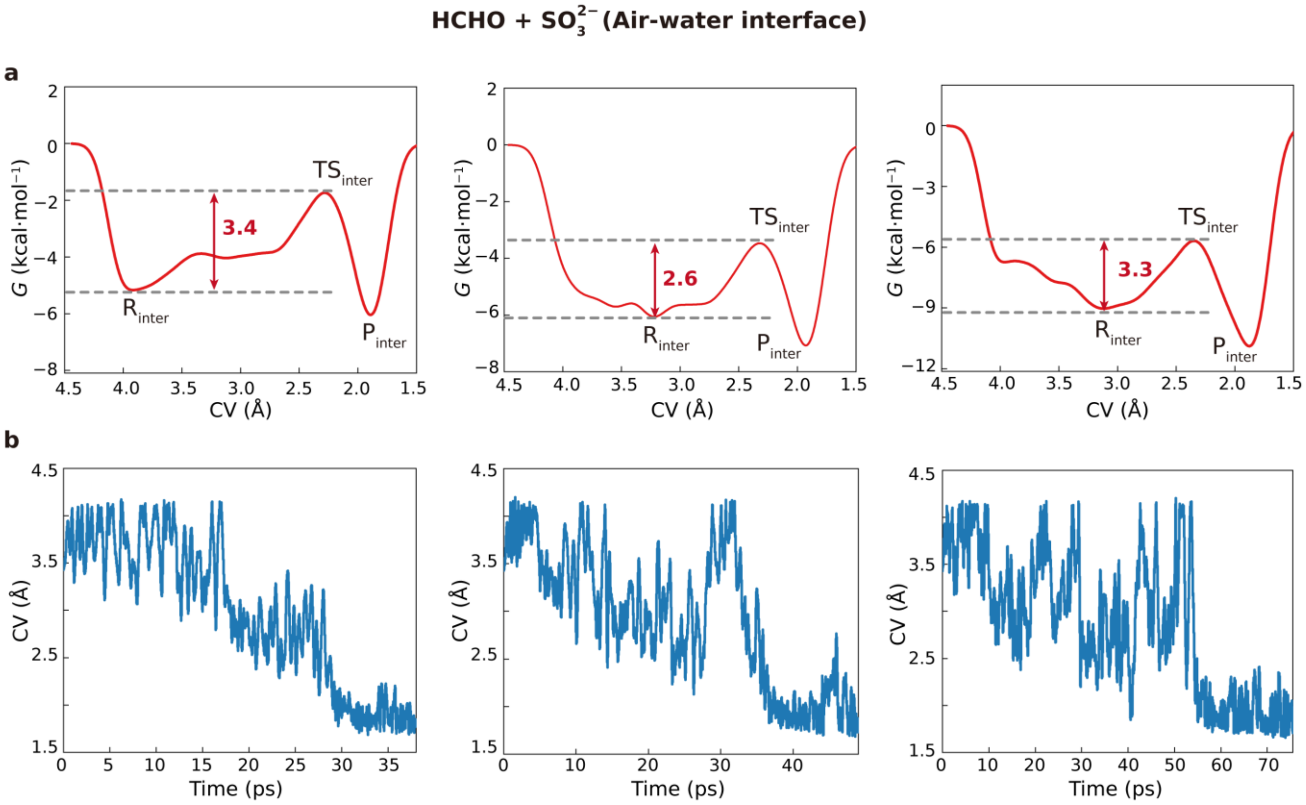

Supplementary Figure 9. Metadynamics-biased results for pathway 3 at the air-water interface. **(a)** Gibbs free-energy profiles as a function of CV changes. **(b)** CV changes as a function of simulation time for three independent simulations.

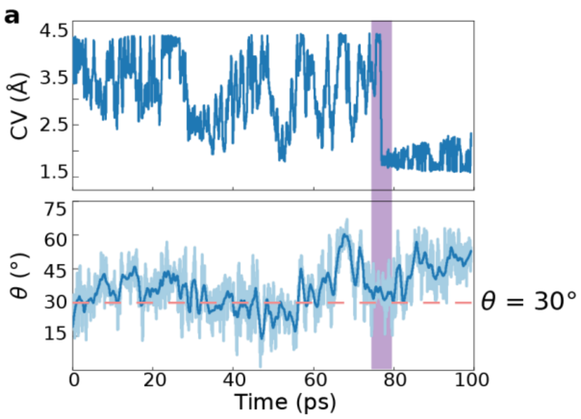

Supplementary Figure 10. (a) Variation of the CV (Top) and the angle between the S−C direction vector and the *z*-axis (Bottom) as a function of the simulation time. The purple shaded area represents the transition state, the light blue area represents the angle for every frame, the dark blue line in the bottom figure is the smoothed value (2500 points) to clarify the trend. **(b)** Snapshot of the $\theta = 37.09°$ at the air-water interface in 77.17 ps.

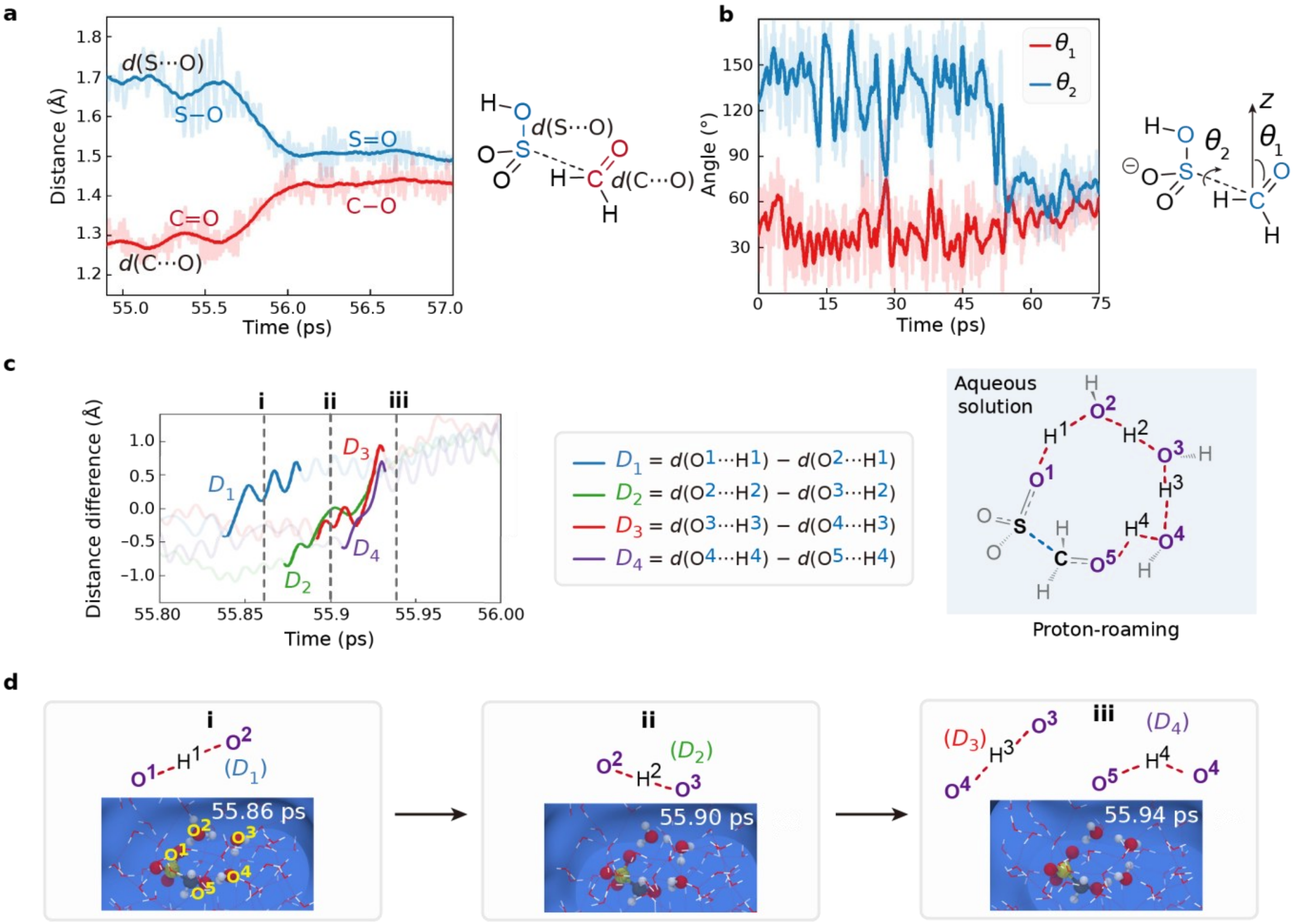

Supplementary Figure 11. (a) Variation of C···O (red line) and S···O (blue line) distances as a function of the simulation time. Shaded area is the distance of every frame, bold lines is the smoothed value (500 points) to clarify the trend. **(b)** Variation of the angle between the C=O direction vector and the *z*-axis (θ_1, 0–180°, red line) and the dihedral angle between C=O direction vector and the S−OH direction vector (θ_2, 0–180°, blue line) as a function of the simulation time. Shaded area is the angle of every frame, bold lines is the smoothed value (2000 points) to clarify the trend. **(d)** (Left) Relevant O···H distance differences (D_1–D_4) variation as a function of the simulation time. (Right) Schematic representation of the water-mediated proton transfer in bulk solution, the proton-roaming pathway is represented as red dash lines. **(d)** Snapshot of the water-mediated proton transfer extracted from the AIMD trajectories of the of HCHO + $HOSO_2^-$ aqueous reaction.

Supplementary Note 3. Electron density difference analysis of the transition state of reaction 1 at the air water interface.

The electron density difference of the transition state for the interface reaction of $HOSO_2^-$ + HCHO is calculated by using Multiwfn program[18]. The cartesian coordinate of the transition state is taken from the AIMD trajectories according to the CV value.

The definition of the electron density difference $\Delta\rho$ is written by equation (1):

$$\Delta\rho = \rho(\mathbf{TS}) - [\rho(\mathrm{HCHO} \cdots \mathrm{HOSO_2^-}) + \rho(\mathrm{slab})] \tag{1}$$

where ρ(**TS**) represents the electron density of the transition state (**TS$_{\mathbf{inter}}$**), ρ(HCHO···$HOSO_2^-$) represents the electron density of the HCHO and $HOSO_2^-$ molecules, and ρ(slab) represents the electron density of all of the water molecules of the TS structure. As shown in **Supplementary Figure 12**, Positive (green) and negative (blue) regions are interspersed with each other, showing that the reactants at the air-water interface is polarized, but no charge transfer occurred at the air-water interface.

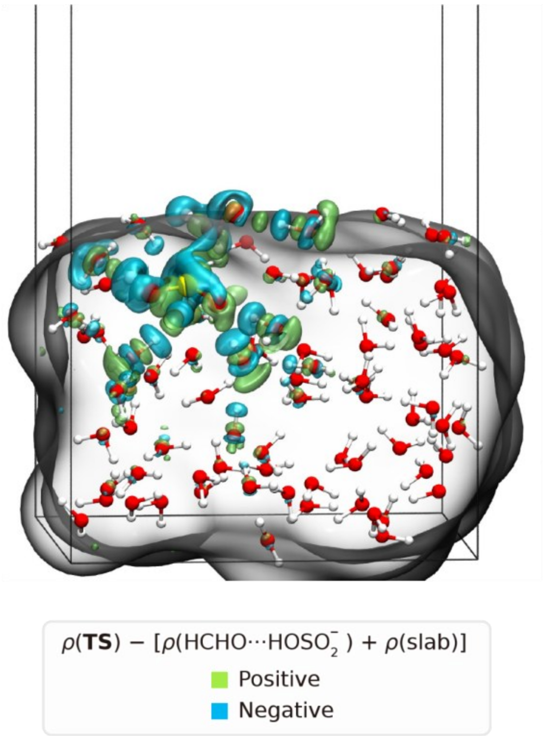

Supplementary Figure 12. Isodensity surface (isodensity = 0.02) of the distribution of the density difference of heterogeneous HCHO + $HOSO_2^-$ reaction. Green denotes regions of positive values and blue regions of negative values.

Supplementary Note 4. Details of quantum chemical calculations for the HCHO + $HOSO_2^-$ + $(H_2O)_n$ (n = 0, 1, 2, 3, 4) reactions, the HCHO + SO_3^{2-} + $(H_2O)_n$ (n = 0, 1, 2, 3, 4) reactions and corresponding wave function analysis.

4.1 Geometrical optimization and the conformational search of global minimum

All geometrical optimizations are obtained at the M06-2x/6-311++G(d,p) level of theory by using Gaussian 16 software[2]. All optimized structures are summarized in **Supplementary Figure 16** and **Supplementary Figure 17** The search of the transition state for the $HOSO_2^-$ + HCHO reaction (see **Supplementary Figure 18**, **TS1**) and the SO_3^{2-} + HCHO reaction (see **Supplementary Figure 19**, **TS2**) are carried out initially. For the transition state of the HCHO + $HOSO_2^-$ + $(H_2O)_n$ (n = 0, 1, 2, 3, 4) reaction (see **Supplementary Figure 18**, **TS1-1** to **TS1-4**), a water molecule is utilized as the participant of proton transfer, the others are spectators. For the transition state of the reaction HCHO + SO_3^{2-} + $(H_2O)_n$ (n = 0, 1, 2, 3, 4) (see **Supplementary Figure 19**, **TS2-1** to **TS2-4**), since there is no proton transfer, all water molecules are spectators.

In order to obtain the global minima of different reactants and the transition state clusters, ABCluster 3.0 program[19,20] is utilized for preliminary search, the rigidmol model based on CHARMM 36 forcefield[21] and the Restrained ElectroStatic Potential (RESP) charge[22] are carried out as parameters.

4.2 High-level quantum chemical calculations

All single point energy for reactants, transition stated and products are obtained at the CCSD(T)/aug-cc-pVTZ level. The workflow of the calculation of the reactant complexes, transition states and products are summarized in **Supplementary Figure 13** to **15**.

4.3 Charge composition analysis (CDA)

We obtain the CDA method[23,24] using Multiwfn program[18] to calculate the fragment orbitals for the transition state of the HCHO + $HOSO_2^-$ + $(H_2O)_n$ (n = 0, 1) reactions. We separated both of the transition state of the two reactions into 2 parts. The one contains $HOSO_2^-$ and another contains HCHO. In the transition state of HCHO + $HOSO_2^-$ + H_2O, the H_2O molecule is placed in the HCHO part according to the electrophilicity index result (see **Fig. 4b**). All relative frontier orbitals are calculated in this study.

Supplementary Note 5. Details of quantum chemical calculations for the HCHO + $HOSO_2^-$ and HCHO + SO_3^{2-} reactions with and without an external electric field of 0.1 V/Å.

5.1 The calculation of the direction of the external electric field

The direction of the external electric field by normalized the dipole vector of the transition states of the HCHO + $HOSO_2^-$ and HCHO + SO_3^{2-} reactions is opposite to the normalized dipole vector, with an amplitude of 0.1 V/Å. All calculated dipole vectors and directions of the electric field are summarized in **Supplementary Figure 17**.

5.2 Calculation the energy barrier by adding an electric field

All Cartesian coordinate positions for input structures of the reactants, transition states and products need to be the same as the calculations without the applied electric field. All geometrical optimizations are obtained at the M06-2x/6-311++G(d, p) level and all single point energies of the stationary points are obtained at the CCSD(T)/aug-cc-pVTZ level.

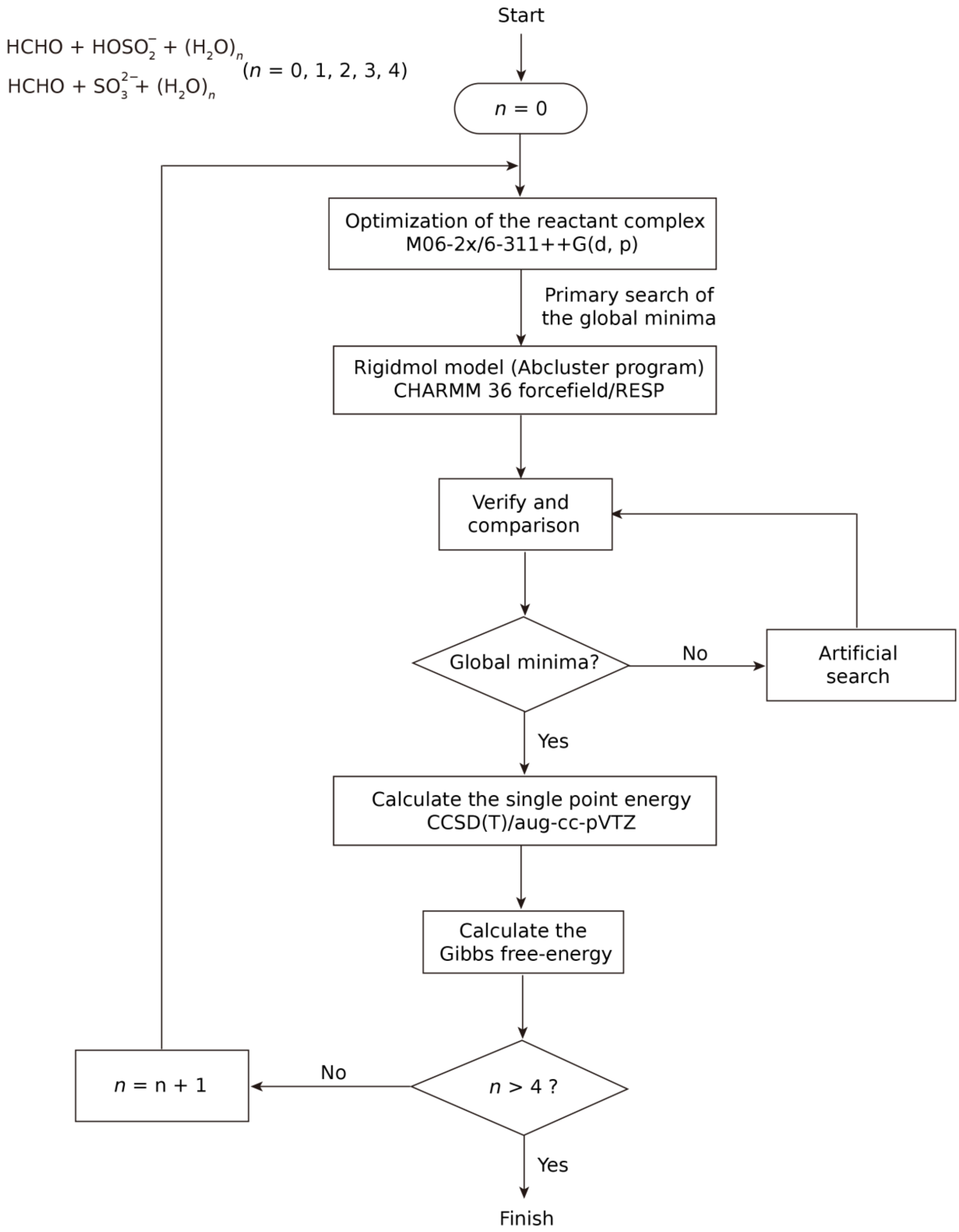

Supplementary Figure 13. Workflow of the high-level quantum chemical calculation of the reaction complexes of the HCHO + $HOSO_2^-$ + (H_2O)$_n$ (n = 0, 1, 2, 3, 4) reaction and the HCHO + SO_3^{2-} + (H_2O)$_n$ (n = 0, 1, 2, 3, 4) reaction.

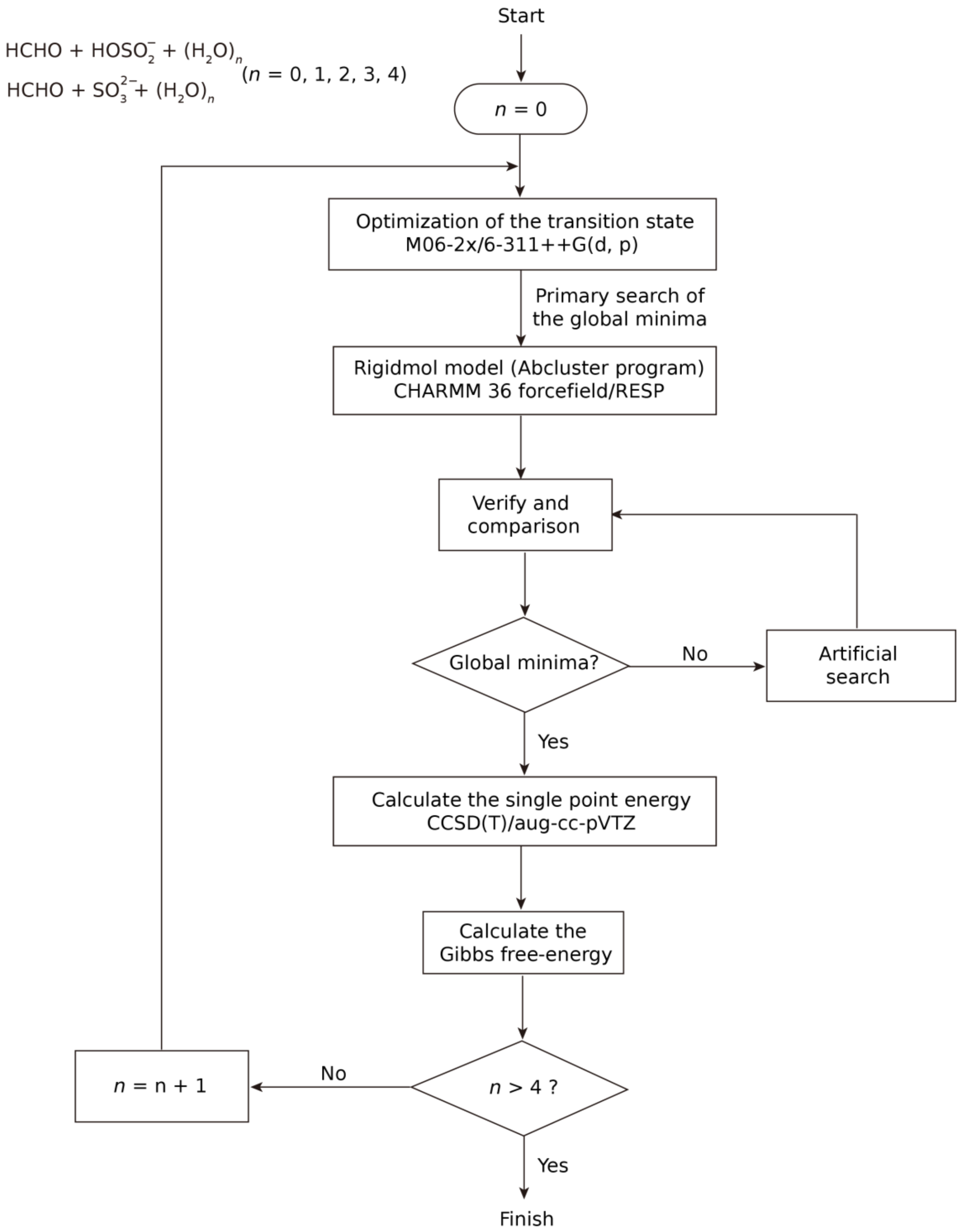

Supplementary Figure 14. Workflow of the high-level quantum chemical calculation of the transition states of the HCHO + HOSO$_2^-$ + (H$_2$O)$_n$ (n = 0, 1, 2, 3, 4) reaction and the HCHO + SO$_3^{2-}$ + (H$_2$O)$_n$ (n = 0, 1, 2, 3, 4) reaction.

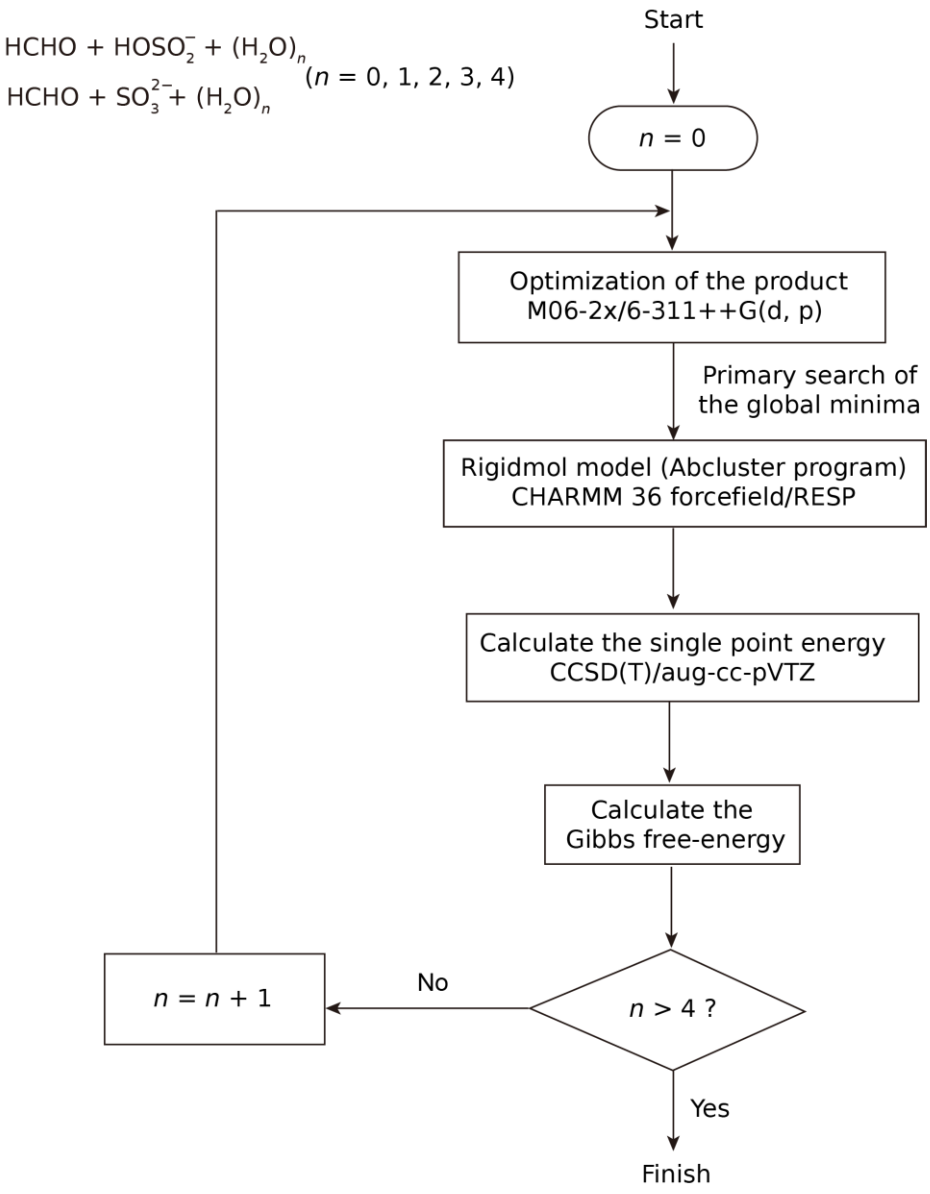

Supplementary Figure 15. Workflow of the high-level quantum chemical calculation of the products of the HCHO + $HOSO_2^-$ + $(H_2O)_n$ (n = 0, 1, 2, 3, 4) reaction and the HCHO + SO_3^{2-} + $(H_2O)_n$ (n = 0, 1, 2, 3, 4) reaction.

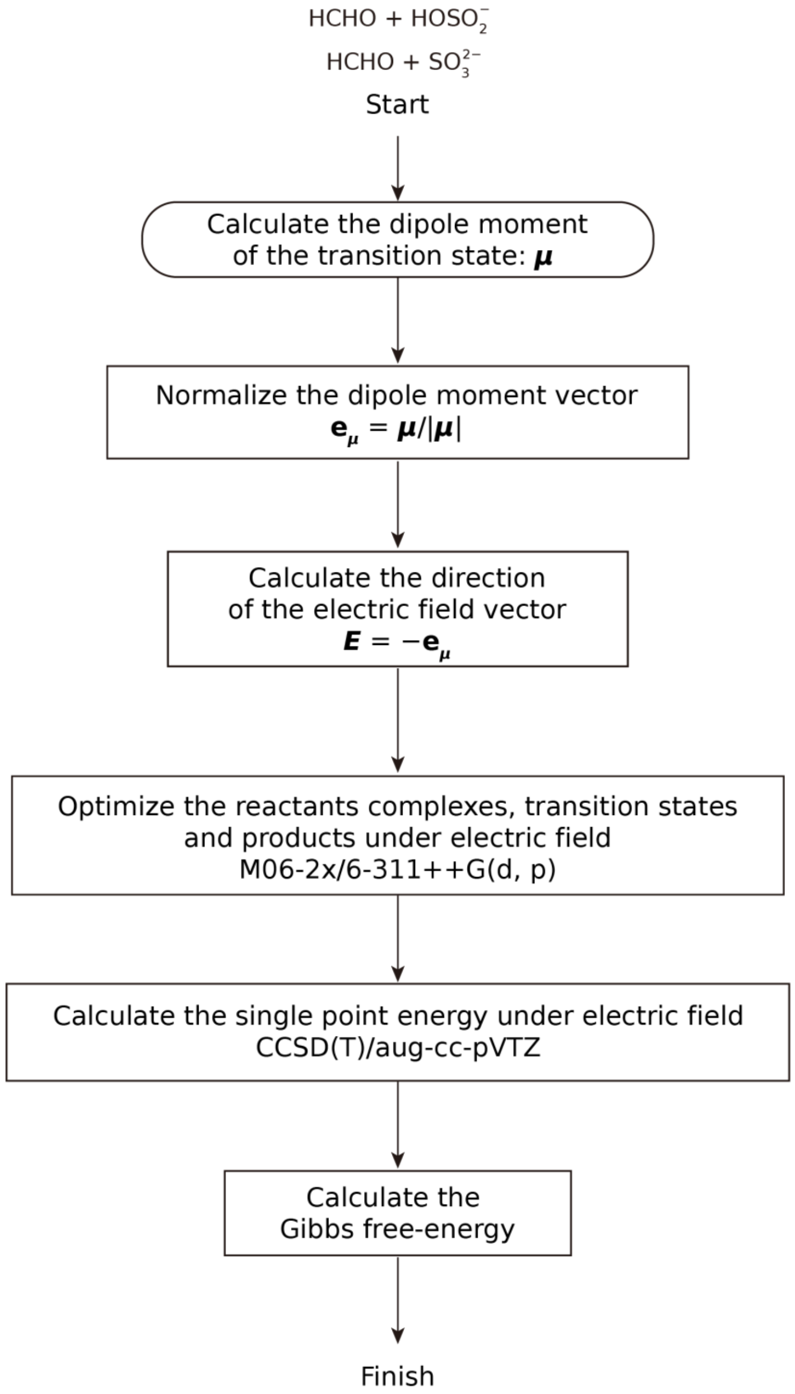

Supplementary Figure 16. Workflow of the High-level quantum chemical calculations for the $HCHO + HOSO_2^-$ and $HCHO + SO_3^{2-}$ reactions with and an external electric field of 0.1 V/Å.

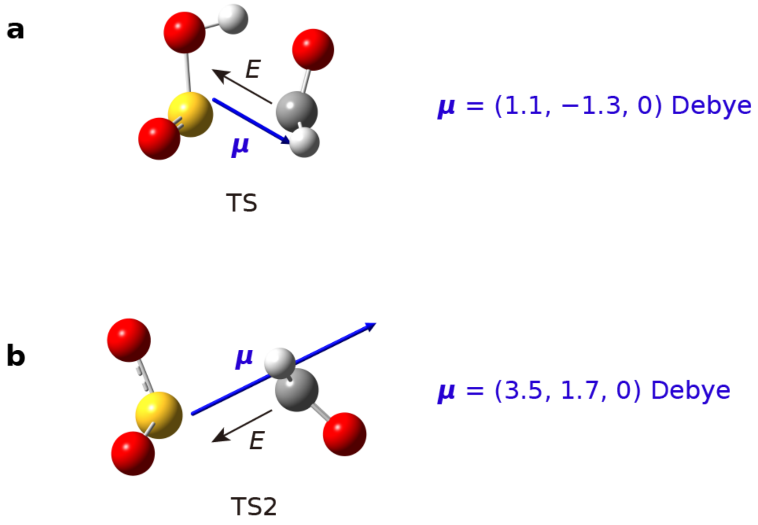

Supplementary Figure 17. The dipole moment vector (blue) and electric field strength vector (black) of transition states for the reactions: **(a)** HCHO + $HOSO_2^-$, **(b)** HCHO + SO_3^{2-}.

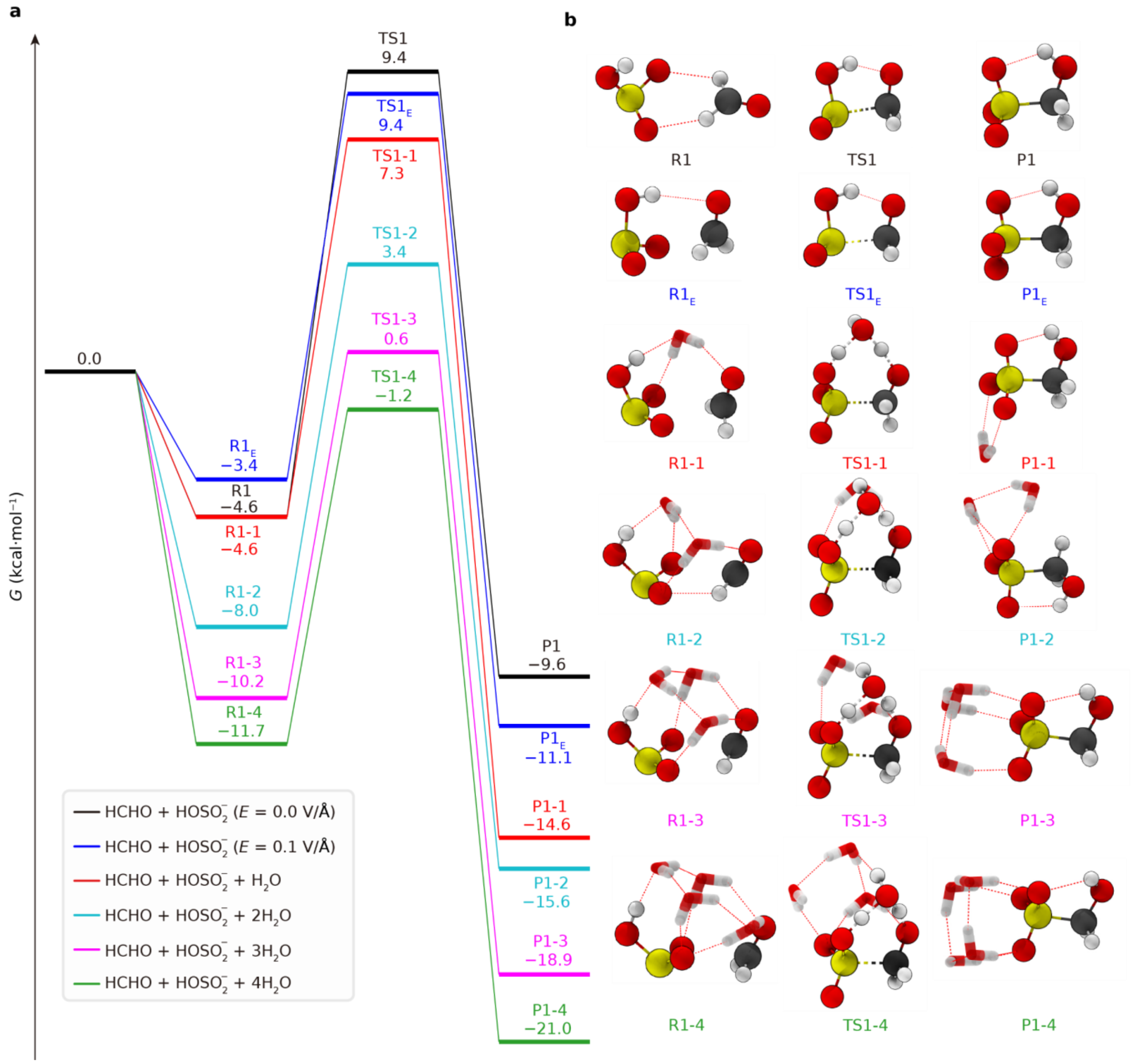

Supplementary Figure 18. (a) Gibbs free-energy profiles of the HCHO + $HOSO_2^-$ reaction with the addition of H_2O molecules (HCHO + $HOSO_2^-$ + $(H_2O)_n$, $n = 0, 1, 2, 3, 4$) and the addition of an external electric field of 0.1 V/Å (**R1$_E$**, **TS1$_E$** and **P1$_E$**). **(b)** Corresponding structures of the stationary points optimized at the M06-2x/6-311++G(d, p) level of theory, relative single point energies of reactant complexes, transition states, and products were obtained at the CCSD(T)/aug-cc-PVTZ level of theory.

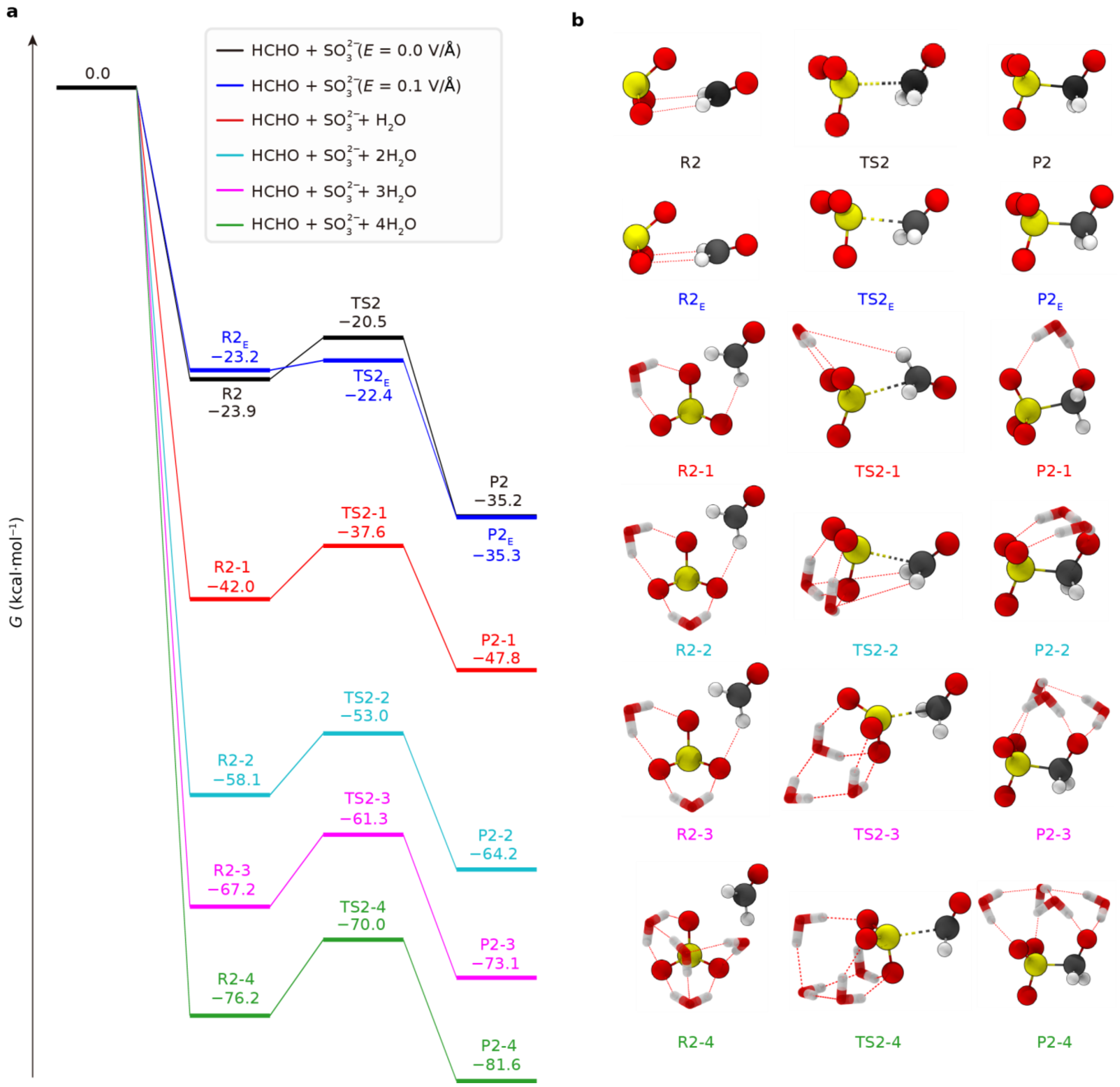

Supplementary Figure 19. (a) Gibbs free-energy profiles of the HCHO + SO_3^{2-} reaction with the addition of H_2O molecules (HCHO + SO_3^{2-} + $(H_2O)_n$, n = 0, 1, 2, 3, 4) and the addition of an external electric field of 0.1 V/Å (**R2**E, **TS2**E and **P2**E). **(b)** Corresponding structures of the stationary points optimized at the M06-2x/6-311++G(d, p) level of theory, relative single point energies of reactant complexes, transition states, and products were obtained at the CCSD(T)/aug-cc-PVTZ level of theory.

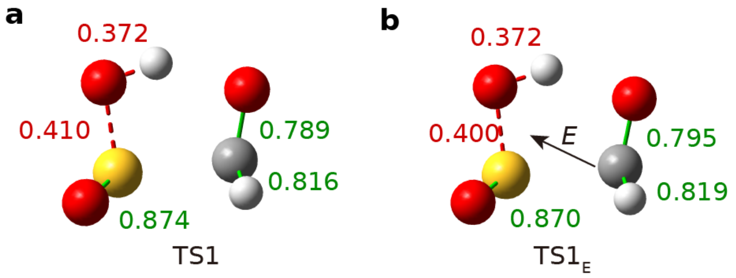

Supplementary Figure 20. Calculated Laplacian bond order of **TS1 (a)** and **$TS1_E$ (b)**.

Supplementary Note 6. Interaction free-energy of HMS-H_2O, HMS-SO_2, and HMS-$HOSO_2^-$ complexes.

Geometrical structure of HMS−H_2O, HMS−SO_2, and HMS−$HOSO_2^-$ complexes are optimized by using Gaussian 16 program[2] with M06-2x functional and 6-311++G(d, p) basis set at polarizable-continuum model (PCM) with water solvent. The definition of interaction free-energy (ΔG_{inter}) is defined by equation (2):

$$\Delta G_{\text{inter}} = G_{\text{HMS-M}} - (G_{\text{HMS}} + G_{\text{M}}) \qquad (2)$$

where M represents the molecule ($H_2O/SO_2/HOSO_2^-$), the optimized structures of HMS, HMS−H_2O, HMS−SO_2, HMS−$HOSO_2^-$ are shown in **Supplementary Figure 21**. The ΔG_{inter} of different complexes are summarized in **Supplementary table 4**.

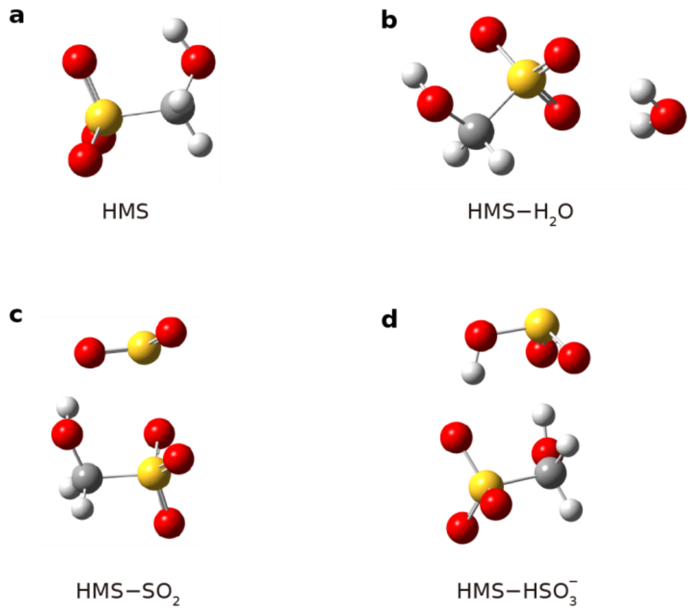

Supplementary Figure 21. Optimized structures of HMS. **(a)**, HMS−H_2O **(b)**, HMS−SO_2 **(c)** and HMS−$HOSO_2^-$ **(d)** with M06-2x/6-311++G(d,p) and PCM model with water solvent.

Supplementary Table 4. Calculated ΔG_{com} of different complexes.

**Complexes**	**ΔG_{com} (kcal/mol)**
HMS−H_2O	−3.2
HMS−SO_2	1.2
HMS−$HOSO_2^-$	−2.0

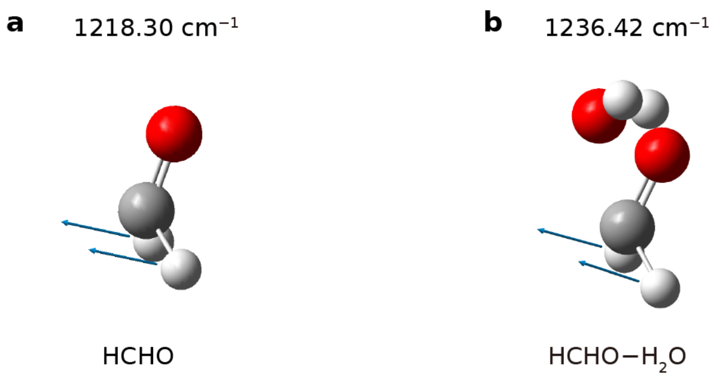

Supplementary Figure 22. Wavenumber (cm^{-1}) of the out-of-phase stretching vibration of the carbonyl group for HCHO **(a)** and HCHO−H_2O **(b)**.

Supplementary Note 7. Details of CMD simulations.

7.1 Preparation of the simulation system

CMD simulations are carried out for the pure water, the HCHO@$(H_2O)_{1000}$ system, the HCHO saturated solution, and a reactant molecule (HCHO/$HOSO_2^-$/SO_3^{2-}) from the bulk water across the air-water interface into the gas phase. All simulation systems use 1000 water molecules as solvent. The structural data of the HCHO and $HOSO_2^-$, SO_3^{2-} molecules were firstly optimized by the Gaussian 16 program with M06-2x/6-311G(d, p) and M06-2x/6-311G++(d, p) level respectively[2-6]. For the aqueous system, the box size was converted from the density of ρ = 997 kg/m^3 of pure water at 300 K. The simulation box size and the details of the molecule numbers are summarized in **Supplementary Table 5**. Note that in order to balancing the charge of simulation system, we used K^+ as counter ion to keep the simulation system neutral; for the HCHO saturated system, the numbers of HCHO molecule were converted from the solubility of HCHO in water[25] (400 g/L).

7.2 Details of CMD parameters

The CMD simulations were preformed using the GROMACS[26] package employing periodic boundary conditions in the NVT ensemble with a temperature of 298.15 K controlled using CSVR thermostat[17]. Optimized potentials for liquid simulations[27] (OPLS-AA) were used for reactant molecules (HCHO/$HOSO_2^-$/SO_3^{2-}) and K^+, and the SPC/E model[28] was used for water molecules. The OPLS-AA forcefield parameters of molecules containing H atoms (HCHO/$HOSO_2^-$) are generated by using LigParGen server[29-31], and the forcefield parameters of SO_3^{2-} is fitted by Hessian matrix from the M06-2x/6-311G++(d, p) level by using Autoff program[32]. All atomic charges are using RESP charge generated by Multiwfn program with the wave function of M06-2x/6-311G(d,p) or M06-2x/6-311G++(d, p) level. All initial structures were firstly generated with the Packmol program[8,9] and all GROMACS input files (.gro/.itp/.top) are generated by Sobtop[33] program. Nonbonding interactions were modeled by the Lennard-Jones and Coulomb potentials. The particle-mesh Ewald summation method[34] was used to calculate electrostatic interactions, and a real-space cutoff of 1.0 nm was applied to nonbonded interactions. The LINCS algorithm[35] was to constrain all hydrogen-containing bonds.

7.3 Simulation of HCHO@$(H_2O)_{1000}$ system, pure water, and HCHO saturated solution.

The initial systems were firstly minimized in energy using the steepest descent algorithm and the conjugated gradient algorithm to remove bad constant. Heating starts the system from a low temperature of 0 K and gradually heats up to 298.15 K at a constant volume. Then 100 ps equilibrations were carried out for pre-equilibrium. The simulation time of each system is shown in **Supplementary**

Table 5. Details of the definitions of the angle between the dipole vector and the *x*, *y*, *z* axis, the definition of the autocorrelation function is shown in **Supplementary Note 8** and **Supplementary Note 9**. The variation of the density and of HCHO saturated solution and the molecular number density of HCHO are shown in **Supplementary Figure 23**.

7.4 Umbrella sampling techniques

Umbrella sampling (US) technique is carried out to calculate the Gibbs free-energy change of reactant molecule (HCHO/$HOSO_2^-$/SO_3^{2-}) from the bulk water across the air-water interface into the gas phase. In order to make the pull distance less than half the box length of the z-direction, the z-direction of the simulation box size for US settings is larger than that for the simulation of HCHO@$(H_2O)_{1000}$ system at the air-water interface. As shown in **Supplementary Figure 24**. The pull distance is about 3.1 nm. The reactant molecule is firstly placed at the center of the water region (z = 0). For all simulations, we divided the pull distance into 60 windows, the distance of each window is represented as Δz.

For all windows, the initial coordinate (represent as z_1, z_2, etc.) is pulled during the energy minimization in two steps. Next, 60 windows are pre-equilibrated for 100 ps. The productive simulation for each window is 10 ns, The Weighted Histogram Analysis Method[36] (WHAM) was used to calculate the Gibbs free-energy variation.

Supplementary Table 5. The molecules, the parameters of the simulation box and the simulation time of CMD simulation.

Simulation	Molecules	x_{box} (nm)	y_{box} (nm)	z_{box} (nm)	Simulation Time (ns) [c]
Pure water	1000 H_2O	3.10768	3.10768	3.10768	0.5
[a]$HCHO@(H_2O)_{1000}$	1 HCHO + 1000 H_2O	3.10768	3.10768	3.10768	500
[b]$HCHO@(H_2O)_{1000}$	1 HCHO + 1000 H_2O	3.10768	3.10768	6.21536	500
HCHO saturated solution	240 HCHO + 1000 H_2O	3.37481	3.37481	6.74962	100
HCHO umbrella sampling	1 HCHO + 1000 H_2O	3.10768	3.10768	9.32304	10
$HOSO_2^-$ umbrella sampling	1 $HOSO_2^-$ + 1000 H_2O + 1 K^+	3.10768	3.10768	9.32304	10
SO_3^{2-} umbrella sampling	1 SO_3^{2-} + 1000 H_2O + 2 K^+	3.10768	3.10768	9.32304	10

[a]CMD simulation in bulk solution.

[b]CMD simulation at t air water interface.

[c]For umbrella sampling, the simulation time represents to the time of each window.

Supplementary Note 8. Definition of the angle between the dipole vector and the *x*(a), *y*(b), *z*(c) axis.

The representative graphical definition of the angle between the dipole vector **μ** (μ_x, μ_y, μ_z) and the *x*, *y* and *z* axis (represented as θ_x, θ_y and θ_z) are shown in **Fig 5e**. In order to consider the direction of the carbonyl, including the oxygen point towards or out of the water slab, we defined these three angles into [0°, 360°). The definitions are given by equation (3):

$$\theta_i = \begin{cases} \frac{180°}{\pi}\arccos\left(\frac{\boldsymbol{\mu}\cdot\mathbf{e}_i}{|\boldsymbol{\mu}|}\right) & (\mu_z > 0) \\ -\frac{180°}{\pi}\arccos\left(\frac{\boldsymbol{\mu}\cdot\mathbf{e}_i}{|\boldsymbol{\mu}|}\right) + 360° & (\mu_z \le 0) \end{cases} \quad (3)$$

where $\mathbf{e}_i$ represent the unit vector of the cartesian axis ($i = x$, y and z). $|\boldsymbol{\mu}|$ represents the modulus of the dipole vector **μ**. The *z* component of the dipole vector μ_z represents the carbonyl direction, where $\mu_z <$ 0 (θ_x or $\theta_y \in [0°, 180°)$, $\theta_z \in [0°, 90°) \cup [270°, 360°)$) means that the oxygen atom points to the water slab, $\mu_z > 0$ (θ_x or $\theta_y \in [0°, 180°)$, $\theta_z \in [90°, 270°)$) means that the oxygen atom points to the air. As shown in **Fig. 5e**, due to the hydrophilic of the O atom in the carbonyl, the distribution of *x* and *y* axis is asymmetric between top and bottom, and the distribution of *z* axis is asymmetric between left and right.

Supplementary Note 9. Details of the calculation of the dipole autocorrelation function.

The definition of the autocorrelation function of dipole is given by equation (4):

$$C(t) = \frac{1}{N}\sum_{i=1}^{N}\frac{\langle \boldsymbol{\mu}_i(t)\cdot\boldsymbol{\mu}_i(0)\rangle}{\langle \boldsymbol{\mu}_i(0)\cdot\boldsymbol{\mu}_i(0)\rangle} \tag{4}$$

where $\boldsymbol{\mu}_i(t)$ represents the time evolution of the normalized dipole vectors of molecule, N is the numbers of the molecules, for the pure water system, $N = 1000$, and for the $HCHO@(H_2O)_{1000}$ system, $N = 1$, t is the simulation time. The calculated $C(t)$ is represented as the solid dots in **Fig. 5d**.

To gain the correlation time for each system, we used the assumed Kohlrausch-Williams-Watts stretched exponential for the long-time relaxation behavior of autocorrelation functions $\phi(t)$, as written by mode coupling theory (MCT)[37-39] :

$$\varphi(t) = Ae^{-\left(\frac{t}{\tau_a}\right)^{\beta}} \tag{5}$$

where τ_a is the correlation time, β is the exponent, A is the fitting parameters. The fitted $\phi(t)$ are represented as the solid line in **Fig. 5d**. The calculated correlation time (4.32 ps) of pure water which demonstrates consistency with findings (4.9 ps in 300 K) of Kumar et al[40].

Supplementary Note 10. Details of the simulation of the HCHO saturated solution at the air-water interface.

The factors influencing the Gibbs free-energy at the air-water interface can be measured as the differential equation:

$$\mathrm{d}G = -S\mathrm{d}T + V\mathrm{d}p + \sum_{i=1}^{N} \mu_i \mathrm{d}n_i + \sigma \mathrm{d}s \qquad (6)$$

The first and the second terms of the equation (6) is the influence of the entropy effect, where *G* is the Gibbs free-energy of the given system, *S* represent the entropy, *T* is the temperature, *V* represent the system volume, *p* is the pressure. Considering the transferring process of one particle from the bulk water to the air-water interface for different solute. As shown in **Fig. 5a** and **Supplementary Figure 25**, the hydrophilicity or hydrophobicity of different groups, result in the different configurations of the surrounding water molecules[41], which also lead to the variation of the entropy of the system. For example, comparing the free-energy profile between SO_3^{2-} and $HOSO_2^-$, the more hydrophilic SO_3^{2-} results in a larger energy increase at the air-water interface. For HCHO, the energy trap at the air-water interface is due to the existence of the hydrophobic $-CH_2-$ and the hydrophilic carbon oxygen, which results in the surface water reorientation[42].

According to the Boltzmann distribution:

$$\frac{P(i)}{P(j)} \propto e^{\frac{\varepsilon_j - \varepsilon_i}{\mathrm{k_B}T}} = e^{\frac{N_\mathrm{A}(\varepsilon_j - \varepsilon_i)}{\mathrm{R}T}} \qquad (7)$$

where *P*(*i*) and ε_i represents and is the probability and the molecular energy of the system being in state *i*, N_A is the Avogadro constant. As shown in **Fig. 5a**, by substituting the relative Gibbs free-energy into the equation values as $N_\mathrm{A}\varepsilon_\mathrm{bulk} = 0$ and $N_\mathrm{A}\varepsilon_\mathrm{inter} = -1.1$ kcal/mol, $RT = 0.6$ kcal/mol the ideal state distribution ratio *P*(inter)/ *P*(bulk) in the HCHO saturated solution is 6.3.

However, as shown in the third and the fourth terms of equation (6), where μ_i represents the chemical potential of solute particle, n_i is the particle number, σ represents the surface tension and *s* is the surface area, the influence of the chemical potential and the surface tension should be considered when the solution is saturated. In the saturate solution, the surface tension has undergone substantial changes. Furthermore, the increment of the surface chemical potential could inhibit the solute particle transfers from the bulk water to the air-water interface. These factors result which leads to a lower distribution ratio in the simulation of HCHO saturate solution. Results from our simulation of HCHO saturated solution (see **Supplementary Figure 23**) show that the surface numerical density of HCHO molecule is ~2.2 folds higher than that in the bulk water.

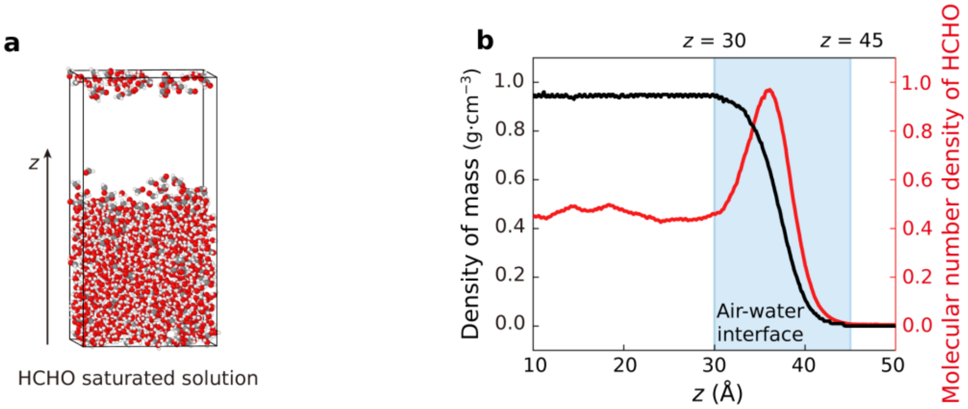

Supplementary Figure 23. (a) The schematic plot of the system for simulating the HCHO saturated solution at the air-water interface. **(b)** The density of mass (black line) of the HCHO saturated solution and the molecular number density of HCHO (red line) as a function of *z*-position.

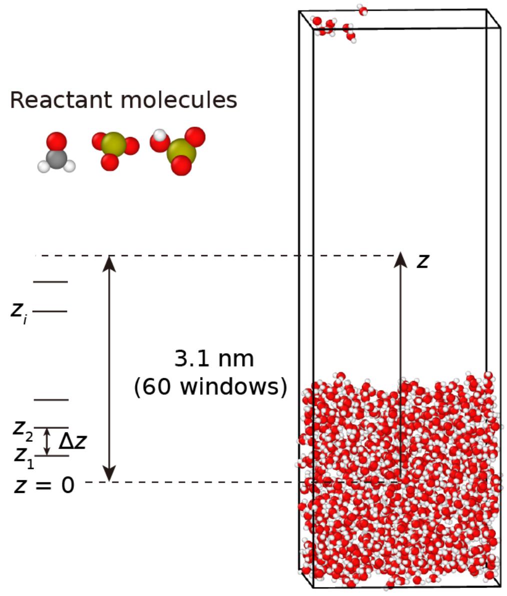

Supplementary Figure 24. The schematic plot of windows the US techniques.

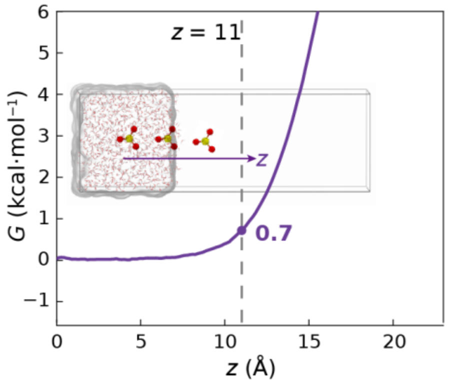

Supplementary Figure 25. Gibbs free-energy profile and the schematic plot of the system for calculating a SO_3^{2-} molecule from the bulk water across the air-water interface into the gas phase.

Supplementary Note 11. Details of the calculation of the activation entropy of the proton transfer.

The quantitative mechanism of the influence of the reaction barrier in bulk solution in comparison with gaseous reaction can be inferred as equation (8).

$$\Delta\Delta G^{\ddagger}_{\mathrm{b-g}} = \Delta G^{\ddagger}_{\mathrm{bulk}} - \Delta G^{\ddagger}_{\mathrm{gas}} = \Delta E_{\mathrm{sol}} + \Delta E_{\mathrm{pol}} + \Delta E_{\mathrm{pro}} \quad (8)$$

Here, $\Delta\Delta G^{\ddagger}_{\mathrm{b-g}}$ represent the difference between the activation Gibbs free-energy in bulk solution and gas phase (−2.6 kcal/mol), ΔE_{sol}, ΔE_{pol}, and ΔE_{pro} represent the activation energy contribution by solvation, polarization and proton-roaming. According to the RxDFT calculations, ΔE_{sol} and ΔE_{pol} equal to 5.0 kcal/mol and −2.1 kcal/mol, respectively. Therefore, from equation (8), the ΔE_{pro} value from our theoretical investigations equals to −5.5 kcal/mol.

Meanwhile, the experimental value of ΔE_{pro} (represented as $\Delta E^{\mathrm{exp}}_{\mathrm{pro}}$) can be estimated by the reaction activation entropy data by Boyce *et al.*[43]. Considering the transition state (**TS**) of HCHO + $HOSO_2^-$ (**Fig. 2d**, **TS$_{\mathbf{bulk}}$**) and HCHO + SO_3^{2-} (**Fig. 6b**, **TS$_{\mathbf{bulk}}$**) reactions in bulk solution from our AIMD simulations. Beyond the common properties of nucleophilic attack form S to C observed in both two **TS**s, the **TS** of HCHO + $HOSO_2^-$ reaction also exhibit distinctive proton-roaming feature. Owing to the fact that the nucleophilic attack and the proton roaming process are nearly independent. The reaction activation entropy for the HCHO + $HOSO_2^-$ reaction ($\Delta S^{\ddagger}_1$) can be estimated as:

$$\Delta S^{\ddagger}_1 \approx \Delta S^{\ddagger}_{\mathrm{nuc}} + \Delta S^{\ddagger}_{\mathrm{pro}} \quad (9)$$

where $\Delta S^{\ddagger}_{\mathrm{nuc}}$ represents the contribution of nucleophilic addition and $\Delta S^{\ddagger}_{\mathrm{pro}}$ represents the contribution of proton-roaming. Meanwhile, the $\Delta S^{\ddagger}_{\mathrm{nuc}}$ is approximately equal to the reaction activation entropy for the HCHO + SO_3^{2-} reaction ($\Delta S^{\ddagger}_2$):

$$\Delta S^{\ddagger}_2 \approx \Delta S^{\ddagger}_{\mathrm{nuc}} \quad (10)$$

Therefore, the equation can be inferred as:

$$\Delta E^{\mathrm{exp}}_{\mathrm{pro}} \approx T(\Delta S^{\ddagger}_1 - \Delta S^{\ddagger}_2) \quad (11)$$

By substituting $\Delta S^{\ddagger}_1$ = −108.0 J/(mol·K), $\Delta S^{\ddagger}_2$ = −31.7 J/(mol·K) by Boyce *et al.*[43], T = 298.15 K, the $\Delta E^{\mathrm{exp}}_{\mathrm{pro}}$ value is −5.4 kcal/mol, which is consistent with our theoretical investigations.

Supplementary Note 12. Investigation of the influence of inorganic ion.

The metadynamics-biased AIMD simulation were performed to investigate the reaction mechanism between bisulfite ($HOSO_2^-$) and HCHO in the presence of Na^+ and Cl^- ions (**Supplementary Figure 26**). The simulation was conducted using a cubic periodic boundary box (1.42 × 1.42 × 1.42 nm^3), containing one Na^+ ion, one Cl^- ion, and 93 water molecules. The collective variable (CV) was defined as the distance between the sulfur atom of $HOSO_2^-$ and the carbon atom of HCHO. The simulation system was carried out in the canonical (NVT) ensemble at the room temperature of 298.15 K, maintained via the velocity rescaling thermostat (CSVR) method. The time step for the simulation was set to 1 fs.

The representative snapshots from the AIMD trajectory (**Supplementary Figure 26a**) indicate that the inclusion of the inorganic ion does not alter the nucleophilic addition mechanism between $HOSO_2^-$ and HCHO relative to the ion-free aqueous environment (see **Fig. 2d** in the manuscript). The reaction still proceeds via a stepwise pathway involving the nucleophilic attack followed by the proton transfer, culminating in the formation of HMS. The CV variation (blue lines in **Supplementary Figure 26c**) delineates the progression through the reactant state (**R_{ion}**), the transition state (**TS_{ion}**), and the final state (**P_{ion}**), corresponding to the structural evolution in **Supplementary Figure 26a**. Furthermore, the Na^+ ion (purple ball) remains solvated by six water molecules (licorice style), forming a well-defined first hydration shell. The distance (D_{ion}) between the Na^+ ion and the center-of-mass (COM) of reactants, illustrated by the purple line in **Supplementary Figure 26c**, fluctuates around 5.5–8 Å, with a spatial separation of approximately two hydration layers. These observations reveal that Na^+ ion acts as a non-interacting spectator, exerting only indirect solvation-mediated effects on the reaction free-energy profile.

From the view of the chemical dynamics, Zhang *et al.*[44] reported a quantitative relationship between the reaction rate constant of the HMS formation and the ionic strength in bulk solution. At low ionic concentrations, the relationship is expressed by equation (12):

$$\lg k = \frac{17.59I}{8.52 + I} + 0.57 \tag{18}$$

where I represents the ionic strength and k denotes the reaction rate constant. The ionic strength is calculated by the following formula:

$$I = \frac{1}{2}\sum c_i z_i^2 \tag{19}$$

in which c_i is the molar concentration (mol/L) of the ion i and z_i is its charge. For the reaction in pure water, the ionic strength (I_{bulk}) is effectively zero. In contrast, for the salt solution in this simulation

system, the salt concentration is equal to 0.577 mol/L, yielding the ionic strength (I_{ion}) of 0.29 mol/L. Substituting into the equation (12), the ratio of the rate constant between the pure water and salt solution ($k_{\text{bulk}}/k_{\text{ion}}$) is calculated to be 0.264. Combining this value with the Eyring equation:

$$k = \frac{\kappa \mathrm{k_B} T}{\mathrm{h}} e^{-\frac{\Delta G^{\ddagger}}{\mathrm{R}T}} \quad (2)$$

where the rate constant is determined by the activation energy $\Delta G^{\ddagger}$, κ is the transmission coefficient, k_B is the Boltzmann constant, T is the temperature, R is the universal gas constant, and h is the Planck constant. Based on this formalism, the difference in the Gibbs free-energy barrier ($\Delta G^{\ddagger}_{\text{bulk}} - \Delta G^{\ddagger}_{\text{ion}}$) can be calculated to be approximately 0.8 kcal/mol. As shown in **Supplementary Figure 26b**, the $\Delta G^{\ddagger}_{\text{ion}}$ of the salt solution system is determined to be 10.2 kcal/mol, while the $\Delta G^{\ddagger}_{\text{bulk}}$ of the pure water system is ~11.4 kcal/mol, yielding an energy barrier difference of 1.2 kcal/mol. The close agreement between theoretical predictions and experimental estimations substantiates the proposed reaction mechanism and underscores the role of ionic strength in modulating reaction kinetics.

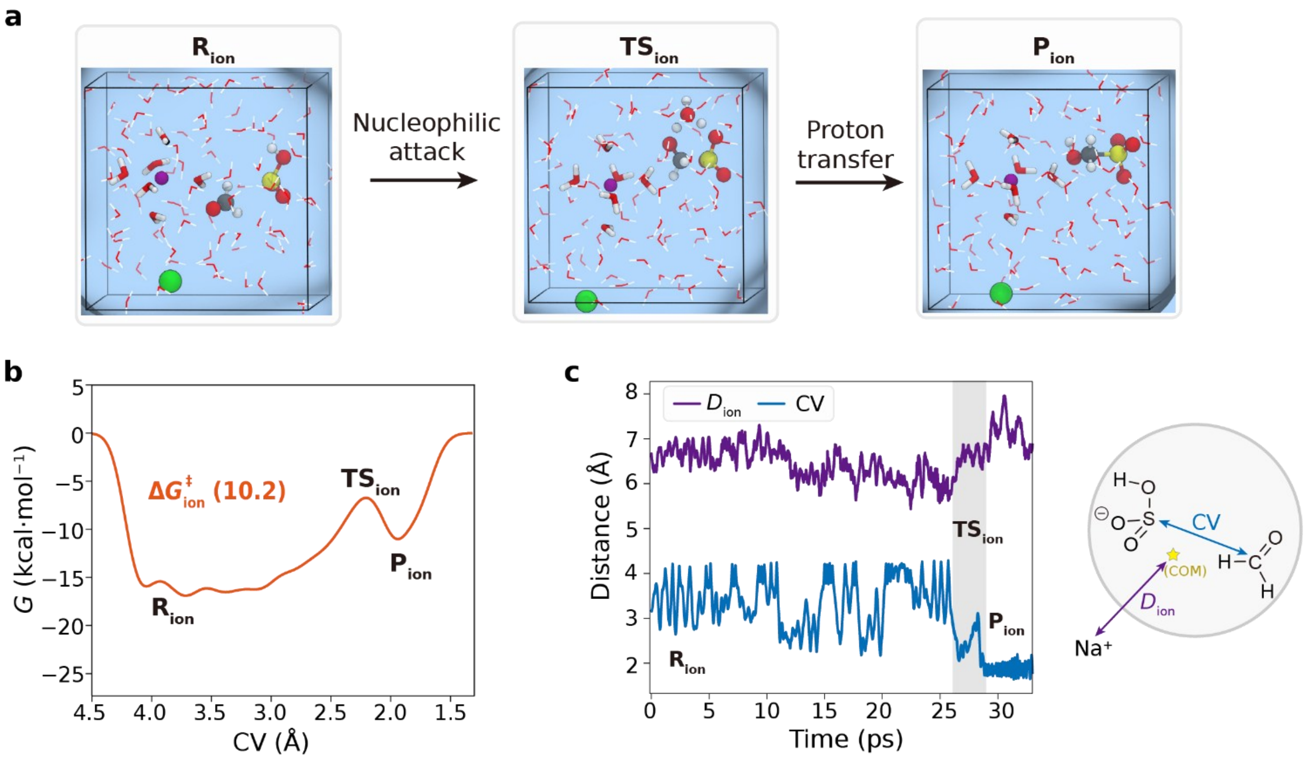

Supplementary Figure 26. **The reaction between $HOSO_2^-$ and HCHO in the bulk salt solution. (a)** Snapshot structures (reactant, **R$_{\mathbf{ion}}$**, transition state, **TS$_{\mathbf{ion}}$** and product, **P$_{\mathbf{ion}}$**) obtained from the metadynamics-biased AIMD simulations. The purple ball indicates the Na^+ ion and the green ball represents the Cl^- ion. The water molecules coordinated to the Na^+ ion are marked as the licorice style for clarity. **(b)** Gibbs free-energy profile as a function of collective variable (CV). **(c)** (Left) Temporal evolution of the distance between the Na^+ ion and the COM (star) of reactants (D_{ion}, purple), along with the variation of the CV (blue). (Right) Schematic definition of D_{ion} and the CV.

Supplementary Note 13. Investigation of the reactivity of sulfonate.

At extreme acidic conditions, sulfonate (HSO_3^-) becomes the predominant species at the air-water interface[45], and its reactivity of HSO_3^- with HCHO is presented in **Supplementary Figure 27**. The electrostatic potential map of HSO_3^-, as shown in **Supplementary Figure 27a**, reveals a pronounced negative potential (blue region) around the oxygen of HSO_3^-, signifying that the oxygen of HSO_3^- serves as the primary nucleophilic site. In contrast, the hydrogen atom bonded to the sulfur atom carries a partial positive charge, indicating a propensity for the proton transfer from the sulfur-bound H to electrophilic centers such as the carbonyl oxygen of HCHO. Frontier molecular orbital analysis reveals that the highest occupied molecular orbital (HOMO) of HSO_3^- is predominantly localized on the oxygen atom, reinforcing the nucleophilic character of the lone pair *p* electron on the oxygen atom.

In conjunction with the electronic structure analysis of HCHO in **Figs. 4b** and **4c** of the manuscript, the nucleophilic addition mechanism of between the HSO_3^- and the HCHO is illustrated in **Supplementary Figure 27c**. The reaction proceeds via a two-step mechanism: the nucleophilic addition from the oxygen in HSO_3^- to the carbonyl in HCHO, followed by the intramolecular proton transfer. The resulting product is an isomer of HMS (HMSi). **Supplementary Figure 27d** depicts the gaseous reaction of HSO_3^- + HCHO, where the reactant needs to overcome a free-energy barrier of $\Delta G^{\ddagger} = 11.3$ kcal/mol to form an intermediate (**INT**). For transition state (**TS**), HSO_3^- and HCHO forms a five-membered ring stabilized by both the hydrogen bonding [H(HSO_3^-)···O(HCHO)] and the nucleophilic interaction [S(HSO_3^-)···C(HCHO)]. This arrangement facilitates the intramolecular proton transfer from the sulfur-bound hydrogen to the carbonyl oxygen of HCHO. However, the significant ring strain associated with this geometry of TS results in a relatively high free-energy barrier of $\Delta G^{\ddagger} = 53.6$ kcal/mol.

To examine the interfacial reactivity, the free-energy profile of HCHO + HSO_3^- reaction at the air-water interface was obtained using the thermodynamic integration (TI)-AIMD method. Results in **Supplementary Figure 27e** show that the free-energy barrier ($\Delta G^{\ddagger}_{sul}$) of the HCHO + HSO_3^- reaction at the air-water interface is 18.1 ± 1.0 kcal/mol. The snapshot structures of the interfacial HCHO + HSO_3^- reaction are provided in **Supplementary Figure 27f**.

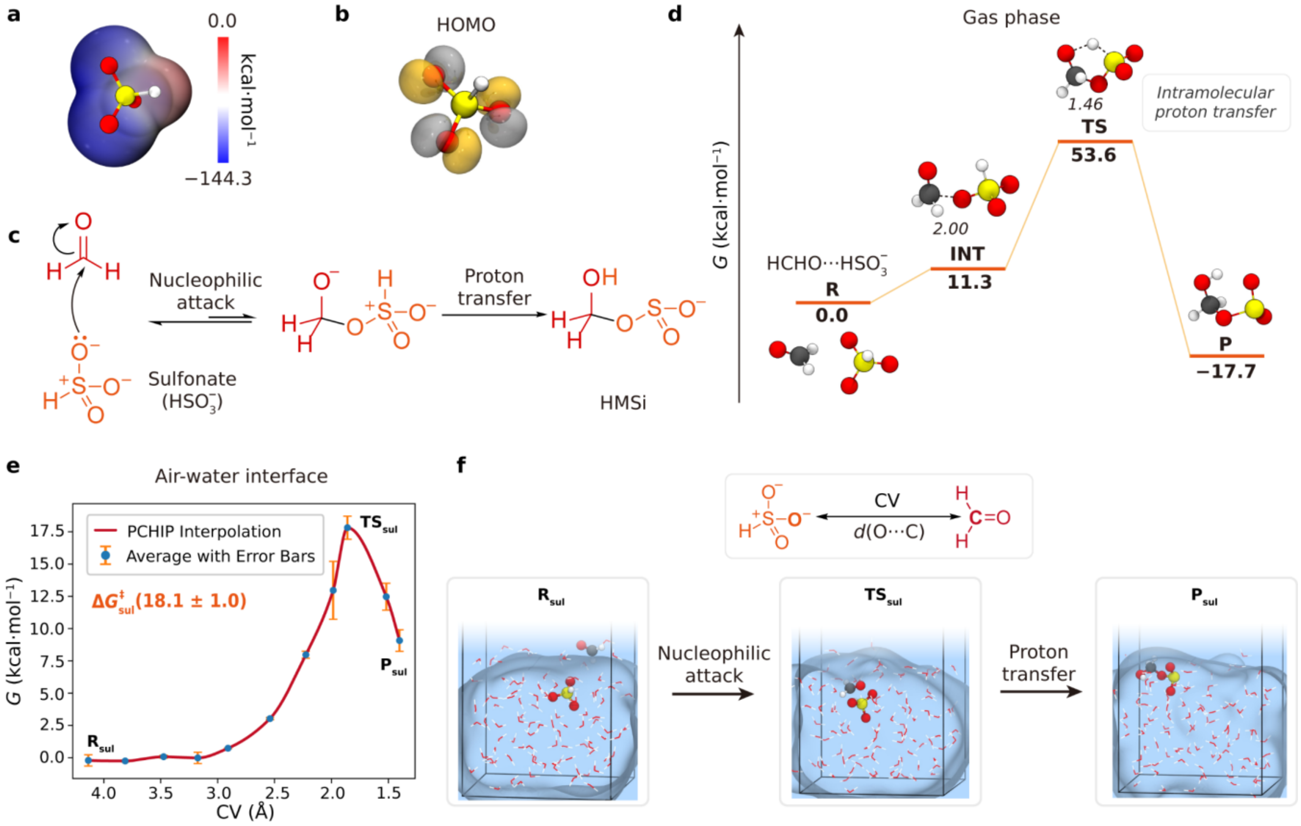

Supplementary Figure 27. Accelerated reactivity of the sulfonate (HSO_3^-) to form the HMSi formation at the air-water interface. (a) Electrostatic potential surface of HSO_3^-. Red regions denote positive electrostatic potential and blue regions of negative potential. **(b)** Isovalue surfaces of the highest occupied molecular orbital (HOMO) of the HSO_3^- (isovalue = ±0.005). **(c)** Representative reaction mechanism for HCHO + HSO_3^- reaction, where the HSO_3^- functions as the nucleophile to attack the carbonyl group. **(d)** Gibbs free-energy profiles for HCHO + HSO_3^- reaction in gas phase with their corresponding optimized structures of the stationary points (**R**, **INT**, **TS** and **P**) at the M06-2x/6-311++G(d,p) level of theory. **(e)** Gibbs free-energy profile of the HCHO + HSO_3^- reaction at the air-water interface obtained from TI-AIMD simulation with the average values of each window (blue dots) for three independent simulations, relevant error bars (colored orange) of each window are calculated using the standard deviation of the corresponding free-energy values of three production runs. The free-energy profile (red line) is generated by Piecewise Cubic Hermite Interpolating Polynomial (PCHIP) interpolation of calculated average free-energy values of three production runs for each window. **(f)** (Top) Definition of the CV for the TI-AIMD simulation. (Bottom) Snapshot structures (reactant, **R$_{sul}$**, transition state, **TS$_{sul}$** and product, **P$_{sul}$**) obtained from the TI-AIMD simulations.

Supplementary Note 14. Influence of Aerosol Acidity or pH.

Overall, as schematically illustrated in **Supplementary Figure 28**, these results show that at extreme acidic condition (pH = 0.8–1.8) where the sulfonate (HSO_3^-) is the dominant aqueous S(IV) species, the surface-accumulated HCHO can undergo nucleophilic addition with this species to form HMSi.

Supplementary Figure 29 outlines the reaction pathways concerning S(IV) species from weak acidic to neutral organic aerosols (pH > 4). As reported by Buttersack *et al*[45], the concentration of SO_3^{2-} increases significantly at pH > 4, primarily due to the deprotonation of HSO_3^- (pK_{SH} = 6.3) and $HOSO_2^-$ (pK_{OH} = 6.9). In such condition, the formation of HMS is energetically favorable, with low energy barrier of SO_3^{2-} + HCHO reaction both at the air-water interface ($\Delta G^{\ddagger}$ = ~2.7 kcal/mol) and in the bulk solution ($\Delta G^{\ddagger}$ = ~5.8 kcal/mol).

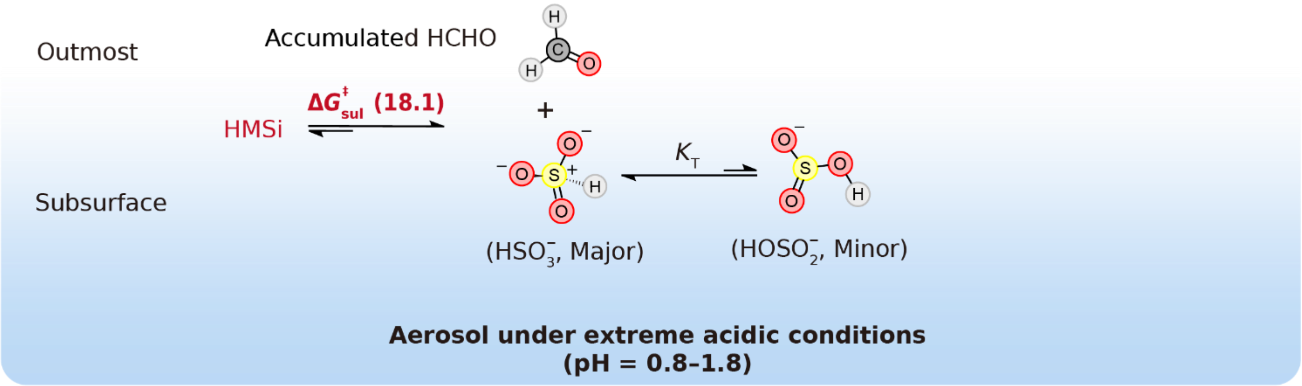

Supplementary Figure 28. Reaction pathways concerning S(IV) species under extreme acidic conditions (pH = 0.8–1.8), the abundant presence of sulfonate (HSO_3^-) enables its reaction with surface-accumulated HCHO to form HMSi.

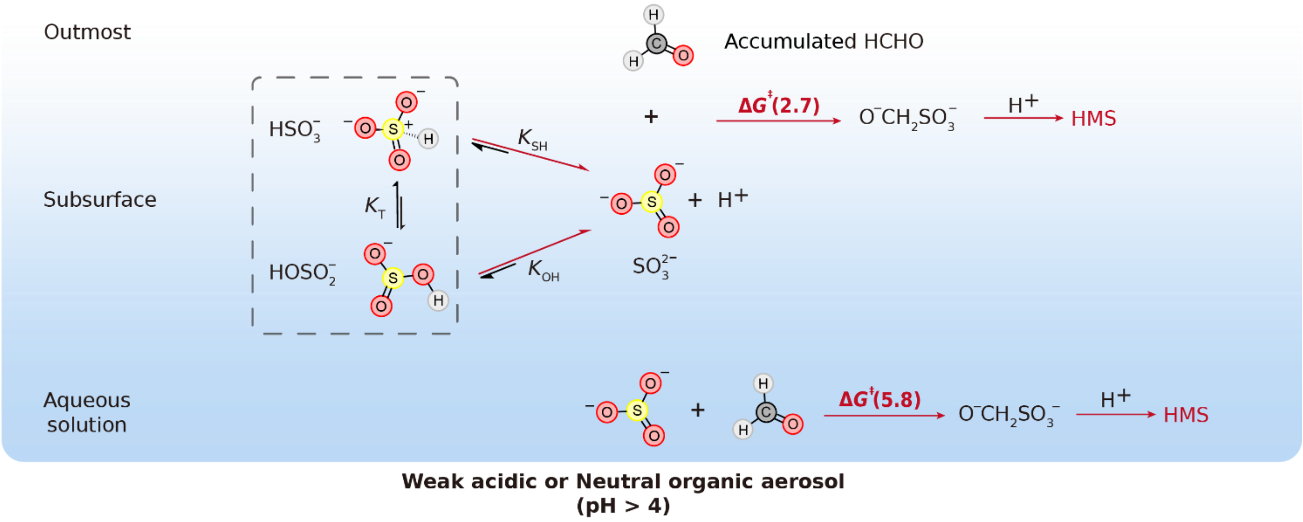

Supplementary Figure 29. Reaction pathways concerning S(IV) species from weak acidic to neutral organic aerosols (pH > 4). The concentration sulfite (SO_3^{2-}) augments due to the deprotonation of the sulfonate (HSO_3^-) and the bisulfite ($HOSO_2^-$). The SO_3^{2-} reacts with HCHO at the air-water interface or in the bulk solution to form the HMS.

Supplementary Note 15. Details of the thermodynamic integration (TI)-AIMD simulation.

The TI-AIMD simulation is employed to investigate the reaction of HCHO + HSO_3^- at the air-water interface and validate the metadynamics results for the reaction between SO_3^{2-} and HCHO in the bulk solution. For the HCHO + HSO_3^- reaction, The CV was defined as the distance between the oxygen atom in HSO_3^- and the carbon atom in HCHO (**Supplementary Figure 26e**). For the HCHO + SO_3^{2-} reaction, The CV was defined as the distance between the oxygen atom in HSO_3^- and the carbon atom in HCHO (**Supplementary Figure 30b**).

For all simulations, a total of 11 sampling windows were implemented to guarantee the smoothness of the calculated free-energy profile. Each window was equilibrated for ~15 ps, followed by a production run of 5 ps for the free-energy sampling. The simulations were performed in the canonical (NVT) ensemble at 298.15 K with temperature control maintained using the velocity rescaling thermostat (CSVR) method. The time step for the simulation was set to 1 fs. The dots in **Supplementary Figure 27e** and **Supplementary Figure 30b** represent the average value obtained from three independent simulations. Relevant error bars of each window (orange) are calculated using the standard deviation of the corresponding free-energy value of three production runs. The free-energy profile is generated by Piecewise Cubic Hermite Interpolating Polynomial (PCHIP) interpolation method of calculated average free-energy values of three production runs.

Concerning the reaction between SO_3^{2-} and HCHO in the bulk solution (**Supplementary Figure 30**), we find that the free-energy barriers obtained from metadynamics (5.8 ± 0.1 kcal/mol) and TI-AIMD simulations ($\Delta G^{\ddagger}_{TI}$ = 6.4 ± 0.7 kcal/mol) are consistent within the margin of error, thereby reinforcing the reliability of the metadynamics simulations. As a result, although the metadynamics simulations likely did not reach convergence due to the lack of recrossing events, the resulting free energy profiles and corresponding conclusions have been supported by TI-AIMD.

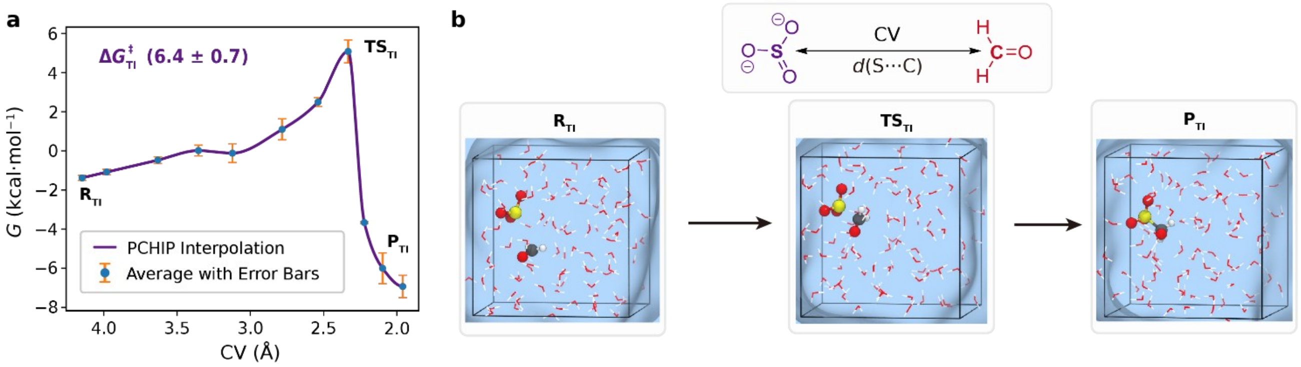

Supplementary Figure 30. TI-AIMD simulation of the reaction between SO_3^{2-} and HCHO in the bulk solution. (a) Gibbs free-energy profile obtained from TI-AIMD simulation with the average values of each window (blue dots) of three independent simulations, relevant error bars (colored orange) of each window are calculated using the standard deviation of the corresponding free-energy values of three production runs. The free-energy profile (purple line) is generated by Piecewise Cubic Hermite Interpolating Polynomial (PCHIP) interpolation of calculated average free-energy values of three production runs. **(b)** (Top) Definition of the CV for the TI-AIMD simulation. (Bottom) Snapshot structures (reactants: **R**$_{TI}$, transition state: **TS**$_{TI}$ and product: **P**$_{TI}$) obtained from the trajectories of TI-AIMD simulation.

Supplementary References

1. Iannuzzi, M., Laio, A. & Parrinello, M. Efficient exploration of reactive potential energy surfaces using Car-Parrinello molecular dynamics. *Phys. Rev. Lett.* **90**, 238302 (2003)
2. Frisch, M. J. et al. *Gaussian 16*, *Revision A.03* (Gaussian, Inc., Wallingford, CT, 2016)
3. Zhao, Y. & Truhlar, D. G. The M06 suite of density functionals for main group thermochemistry, thermochemical kinetics, noncovalent interactions, excited states, and transition elements: two new functionals and systematic testing of four M06-class functionals and 12 other functionals. *Theor. Chem. Acc.* **120**, 215-241 (2008)
4. McLean, A. & Chandler, G. Contracted Gaussian basis sets for molecular calculations. I. Second row atoms, Z= 11–18. *J. Chem. Phys.* **72**, 5639-5648 (1980)
5. Krishnan, R., Binkley, J. S., Seeger, R. & Pople, J. A. Self-consistent molecular orbital methods. XX. A basis set for correlated wave functions. *J. Chem. Phys.* **72**, 650-654 (1980)
6. Clark, T., Chandrasekhar, J., Spitznagel, G. W. & Schleyer, P. V. R. Efficient diffuse function-augmented basis sets for anion calculations. III. The 3-21+ G basis set for first-row elements, Li–F. *J. Comput. Chem.* **4**, 294-301 (1983)
7. Frisch, M. J., Pople, J. A. & Binkley, J. S. Self-consistent molecular orbital methods 25. Supplementary functions for Gaussian basis sets. *J. Chem. Phys.* **80**, 3265-3269 (1984)
8. Martínez, L., Andrade, R., Birgin, E. G. & Martínez, J. M. PACKMOL: A package for building initial configurations for molecular dynamics simulations. *J. Comput. Chem.* **30**, 2157-2164 (2009)
9. Martínez, J. M. & Martínez, L. Packing optimization for automated generation of complex system's initial configurations for molecular dynamics and docking. *J. Comput. Chem.* **24**, 819-825 (2003)
10. Kühne, T. D. et al. CP2K: An electronic structure and molecular dynamics software package-Quickstep: efficient and accurate electronic structure calculations. *J. Chem. Phys.* **152**, (2020)
11. Stewart, J. J. Optimization of parameters for semiempirical methods V: Modification of NDDO approximations and application to 70 elements. *J. Comput. Chem.* **13**, 1173-1213 (2007)
12. Becke, A. D. Density-functional exchange-energy approximation with correct asymptotic behavior. *Phys. Rev. A* **38**, 3098 (1988)
13. Lee, C., Yang, W. & Parr, R. G. Development of the Colle-Salvetti correlation-energy formula into a functional of the electron density. *Phys. Rev. B* **37**, 785 (1988)
14. Grimme, S. Accurate description of van der Waals complexes by density functional theory including empirical corrections. *J. Comput. Chem.* **25**, 1463-1473 (2004)

15. Johnson, E. R. & Becke, A. D. A post-Hartree-Fock model of intermolecular interactions: inclusion of higher-order corrections. *J. Chem. Phys.* **124**, (2006)

16. Goedecker, S., Teter, M. & Hutter, J. Separable dual-space Gaussian pseudopotentials. *Phys. Rev. B* **54**, 1703 (1996)

17. Bussi, G., Donadio, D. & Parrinello, M. Canonical sampling through velocity rescaling. *J. Chem. Phys.* **126**, (2007)

18. Lu, T. & Chen, F. Multiwfn: A multifunctional wavefunction analyzer. *J. Comput. Chem.* **33**, 580-592 (2012)

19. Zhang, J. & Dolg, M. ABCluster: the artificial bee colony algorithm for cluster global optimization. *Phys. Chem. Chem. Phys.* **17**, 24173-24181 (2015)

20. Zhang, J. & Dolg, M. Global optimization of clusters of rigid molecules using the artificial bee colony algorithm. *Phys. Chem. Chem. Phys.* **18**, 3003-3010 (2016)

21. Vanommeslaeghe, K. et al. CHARMM general force field: A force field for drug-like molecules compatible with the CHARMM all-atom additive biological force fields. *J. Comput. Chem.* **31**, 671-690 (2010)

22. Bayly, C. I., Cieplak, P., Cornell, W. & Kollman, P. A. A well-behaved electrostatic potential based method using charge restraints for deriving atomic charges: the RESP model. *J. Phys. Chem.* **97**, 10269-10280 (1993)

23. Dapprich, S. & Frenking, G. Investigation of donor-acceptor interactions: a charge decomposition analysis using fragment molecular orbitals. *J. Phys. Chem.* **99**, 9352-9362 (1995)

24. Xiao, M. & Lu, T. Generalized charge decomposition analysis (GCDA) method. *J. Adv. Phys. Chem.* **4**, 111-124 (2015)

25. Pickrell, J. A., Mokler, B. V., Griffis, L. C., Hobbs, C. H. & Bathija, A. Formaldehyde release rate coefficients from selected consumer products. *Environ. Sci. Technol.* **17**, 753-757 (1983)

26. Abraham, M. J. et al. GROMACS: High performance molecular simulations through multi-level parallelism from laptops to supercomputers. *SoftwareX* **1**, 19-25 (2015)

27. Jorgensen, W. L., Maxwell, D. S. & Tirado-Rives, J. Development and testing of the OPLS all-atom force field on conformational energetics and properties of organic liquids. *J. Am. Chem. Soc.* **118**, 11225-11236 (1996)

28. Berendsen, H.-J.-C., Grigera, J.-R. & Straatsma, T. P. The missing term in effective pair potentials. *J. Phys. Chem.* **91**, 6269-6271 (1987)

29. Jorgensen, W. L. & Tirado-Rives, J. Potential energy functions for atomic-level simulations of water and organic and biomolecular systems. *Proc. Natl. Acad. Sci. U.S.A.* **102**, 6665-6670

(2005)

30. Dodda, L. S., Cabeza de Vaca, I., Tirado-Rives, J. & Jorgensen, W. L. LigParGen web server: an automatic OPLS-AA parameter generator for organic ligands. *Nucleic Acids Res.* **45**, W331-W336 (2017)

31. Dodda, L. S., Vilseck, J. Z., Tirado-Rives, J. & Jorgensen, W. L. 1.14* CM1A-LBCC: localized bond-charge corrected CM1A charges for condensed-phase simulations. *J. Phys. Chem. B* **121**, 3864-3870 (2017)

32. Wang, C. et al. *AuToFF Program,*, *Vesrion 1.0.* (Hzwtech. Shanghai 2023, 2023)

33. Lu, T. *Sobtop*, *1.0(dev5)* http://sobereva.com/soft/Sobtop (access on 2025.1.20)

34. Darden, T., York, D. & Pedersen, L. Particle mesh Ewald: an N·log (N) method for Ewald sums in large systems. *J. Chem. Phys.* **98**, 10089-10092 (1993)

35. Hess, B., Bekker, H., Berendsen, H. J. & Fraaije, J. G. LINCS: A linear constraint solver for molecular simulations. *J. Comput. Chem.* **18**, 1463-1472 (1997)

36. Hub, J. S., De Groot, B. L. & Van Der Spoel, D. g_wham A Free Weighted Histogram Analysis Implementation Including Robust Error and Autocorrelation Estimates. *J. Chem. Theory Comput.* **6**, 3713-3720 (2010)

37. Gallo, P., Sciortino, F., Tartaglia, P. & Chen, S.-H. Slow dynamics of water molecules in supercooled states. *Phys. Rev. Lett.* **76**, 2730 (1996)

38. Starr, F. W., Harrington, S., Sciortino, F. & Stanley, H. E. Slow dynamics of water under pressure. *Phys. Rev. Lett.* **82**, 3629 (1999)

39. Starr, F. W., Sciortino, F. & Stanley, H. E. Dynamics of simulated water under pressure. *Phys. Rev. E* **60**, 6757 (1999)

40. Kumar, P., Franzese, G., Buldyrev, S. V. & Stanley, H. E. Molecular dynamics study of orientational cooperativity in water. *Phys. Rev. E* **73**, 041505 (2006)

41. Chandler, D. Interfaces and the driving force of hydrophobic assembly. *Nature* **437**, 640-647 (2005)

42. Martins-Costa, M. T. C., Anglada, J. M., Francisco, J. S. & Ruiz-Lopez, M. F. Reactivity of volatile organic compounds at the surface of a water droplet. *J. Am. Chem. Soc.* **134**, 11821-11827 (2012)

43. Boyce, S. D. & Hoffmann, M. R. Kinetics and mechanism of the formation of hydroxymethanesulfonic acid at low pH. *J. Phys. Chem.* **88**, 4740-4746 (1984)

44. Zhang, H., Xu, Y. & Jia, L. Hydroxymethanesulfonate formation as a significant pathway of transformation of SO2. *Atmos. Environ.* **294**, 119474 (2023)

45. Buttersack, T. et al. Direct observation of the complex S(IV) equilibria at the liquid-vapor interface. *Nat. Commun.* **15**, 8987 (2024)
